# Delivering a toxic metal to the active site of urease

**DOI:** 10.1126/sciadv.adf7790

**Published:** 2023-04-21

**Authors:** Yap Shing Nim, Ivan Yu Hang Fong, Justin Deme, Ka Lung Tsang, Joseph Caesar, Steven Johnson, Longson Tsz Hin Pang, Nicholas Man Hon Yuen, Tin Long Chris Ng, Tung Choi, Yakie Yat Hei Wong, Susan M. Lea, Kam-Bo Wong

**Affiliations:** ^1^ School of Life Sciences, Centre for Protein Science and Crystallography, State Key Laboratory of Agrobiotechnology, The Chinese University of Hong Kong, Hong Kong, China.; ^2^ Sir William Dunn School of Pathology, University of Oxford, South Parks Road, Oxford OX1 3RE, UK.; ^3^ Center for Structural Biology, CCR, NCI, Boyles Street, Frederick, MD 21702, USA.

## Abstract

Urease is a nickel (Ni) enzyme that is essential for the colonization of *Helicobacter pylori* in the human stomach. To solve the problem of delivering the toxic Ni ion to the active site without diffusing into the cytoplasm, cells have evolved metal carrier proteins, or metallochaperones, to deliver the toxic ions to specific protein complexes. Ni delivery requires urease to form an activation complex with the urease accessory proteins UreFD and UreG. Here, we determined the cryo–electron microscopy structures of *H. pylori* UreFD/urease and *Klebsiella pneumoniae* UreD/urease complexes at 2.3- and 2.7-angstrom resolutions, respectively. Combining structural, mutagenesis, and biochemical studies, we show that the formation of the activation complex opens a 100-angstrom-long tunnel, where the Ni ion is delivered through UreFD to the active site of urease.

## Teaser

Formation of a urease/accessory-protein complex opens a 100-Å long tunnel that facilitates nickel delivery in urease maturation.

## INTRODUCTION

Urease is a virulence factor of *Helicobacter pylori* that infects half of the human population, leading to an increased risk of peptic ulcers and gastric cancer (*1*). The enzyme, hydrolyzing urea into carbon dioxide and ammonia, is essential to the survival of the pathogen in the acidic environment of the human stomach (*2*). Most ureases are nickel (Ni) enzymes containing two Ni(II) ions bridged by a carbamylated lysine residue (Lys^219^ of *H. pylori* urease) in their active sites (*3*–*6*). Metal ions at the top of the Irving-Williams series (*7*) such as Ni(II) ions are toxic because they can displace weaker ions [e.g., Mg^2+^ in guanosine triphosphatase (GTPase)] from the active site of cellular enzymes and can inactivate these enzymes (*8*).

**Fig. 1.**
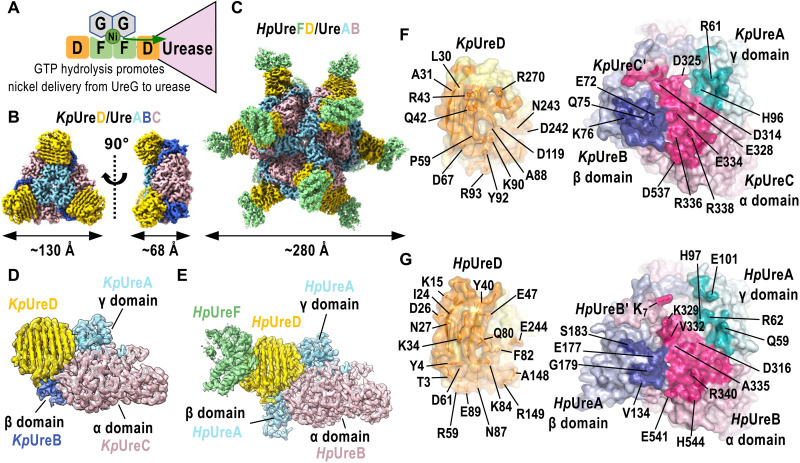
Cryo-EM structures of *Helicobacterpylori Hp*UreFD/urease and *Klebsiellapneumoniae Kp*UreD/urease complexes. (**A**) Ni delivery from UreG to urease requires the formation of the UreFD/urease complex. (**B** to **E**) Cryo–electron microscopy (cryo-EM) maps of the (B) *K. pneumoniae Kp*UreD/urease and (C) *H. pylori Hp*UreFD/urease complexes. Structures of the (D) *Kp*UreD/urease and (E) *Hp*UreFD/urease complexes are overlaid with their corresponding maps. The maps are contoured at 4σ except those of *Hp*UreD (yellow) and *Hp*UreF (green), which are contoured at 2σ and 1σ, respectively. Each catalytic unit of *K. pneumoniae* urease consists of *Kp*UreA (cyan), *Kp*UreB (blue), and *Kp*UreC (pink), representing the γ, β, and α domains. In *H. pylori* urease, *Hp*UreA (cyan) is a fusion protein of the γ and β domains, and *Hp*UreB (pink) is the α domain of the urease. One *Kp*UreD (yellow) or *Hp*UreFD is bound to each catalytic unit of *Kp*UreABC or *Hp*UreAB, respectively. (**F** and **G**) The interacting surfaces between UreD and the urease in the (F) *Kp*UreD/urease and (G) *Hp*UreFD/urease are color-coded in darker shades of orange (UreD), pink (α domain), blue (β domain), and cyan (γ domain). Residues involved in polar contacts are indicated.

The urease maturation pathway represents a paradigm for how cells solve the problem of delivering a toxic metal ion to the active site of an essential enzyme. Cells have evolved metallochaperones, or metal carrier proteins, to deliver the Ni(II) ions from one protein to another via the formation of specific protein-protein complexes so that the toxic metal ions do not diffuse into the cytoplasm (*9*). In the urease maturation pathway, Ni delivery is assisted by four urease accessory proteins UreD, UreE, UreF, and UreG (fig. S1) (*9*–*11*). UreE exists as a homodimer in solution containing the conserved Gly-Asn-Arg-His motif that binds a Ni(II) ion at the dimeric interface (*12*–*14*). UreG is a GTPase that undergoes guanosine triphosphate (GTP)–dependent dimerization that brings the conserved Cys-Pro-His (CPH) motif to the dimer interface to bind a Ni(II) ion in a square planar coordination (*15*). UreD interacts with UreF to form a dimer of heterodimeric UreFD complex, which induces conformational changes in UreF to recruit a UreG dimer to form a UreGFD complex (*16*, *17*). UreE provides the upstream source of Ni(II) ions, which are delivered to UreG via the formation of a UreE_2_G_2_ heterodimeric complex (*15*, *18*, *19*). After receiving its Ni, the Ni-bound UreG dimer forms an activation complex with UreFD and urease, and activates urease upon GTP hydrolysis with the help of UreFD (Fig. 1A) (*15*, *17*, *20*).

How Ni is transferred from UreG to urease remains elusive. The Ni binding site of urease is deeply buried (*3*, *4*), so it is not known how Ni can access the urease active site. Moreover, because UreG and the urease are topologically separated by the UreFD complex, the Ni binding site of UreG will likely be far away from the urease (Fig. 1A). It is also not known what could be the role of the UreFD complex in this process. In this study, we determined the cryo–electron microscopy (cryo-EM) structures of *H. pylori Hp*UreFD/urease complex and *Klebsiella pneumoniae Kp*UreD/urease complex. We show that formation of the UreFD/urease complex opens a tunnel from the buried urease active site that can connect to the Ni binding site in UreG. Further supported by mutagenesis and biochemical studies, we suggest that this tunnel facilitates the delivery of Ni(II) ion from UreG to the urease.

## RESULTS

### Cryo-EM structures of *Hp*UreFD/urease and *Kp*UreD/urease complexes are determined at 2.3- and 2.7-Å resolution

To facilitate purification of urease in complex with urease accessory proteins, Strep-tags were added to the coding sequences of *Kp*UreD and *Hp*UreD, which were coexpressed with other proteins in the urease operon in *Escherichia coli* (see Materials and Methods). The complexes of *Kp*UreD/urease and *Hp*UreFD/urease were purified by Strep-Tactin affinity chromatography, followed by size exclusion chromatography. In our hands, the *H. pylori* urease aggregated when coexpressed with the urease accessory proteins likely because *Hp*UreFD forms a dimer with two urease binding sites that could cross-link urease dodecamers. We have previously shown that dimerization of *Hp*UreFD is disrupted by R179A/Y183D substitutions to *Hp*UreF (*17*). Here, we introduced R179A/Y183D substitutions to *Hp*UreF so that we could obtain discrete particles of *Hp*UreFD/urease complexes for cryo-EM analyses. Additional substitution of E140A to *Hp*UreD was found to stabilize the complex, and protein samples of *H. pylori* urease (*Hp*UreAB) in complex with *Hp*UreF(R179A/Y183D) and *Hp*UreD(E140A) were finally used for data collection.

The EM maps of *Hp*UreFD/urease and *Kp*UreD/urease were solved to 2.3- and 2.7-Å resolution, respectively (Fig. 1 and fig. S2). Cartoon representation and secondary structure assignment of the modeled structures are shown in the figs. S3 to S5. In *K. pneumoniae* urease, each catalytic unit is constituted by the α (*Kp*UreC), β (*Kp*UreB), and γ (*Kp*UreA) domains (Fig. 1D). Similar to the quaternary structure of *Klebsiella aerogenes* urease (*3*), *K. pneumoniae* urease contains three catalytic units arranged in a C3 trimer that resembles a triangular disc-like structure (Fig. 1B). The vertexes of the urease trimer are bound with a *Kp*UreD molecule (Fig. 1, B and D) that has the characteristic β helix fold (fig. S3). In *H. pylori* urease, each catalytic unit is constituted by *Hp*UreA (a fusion of the β and γ domains) and *Hp*UreB (α domain) (Fig. 1E and figs. S4 and S5). Four copies of the urease trimers form a dodecameric quaternary structure with 12 protruding *Hp*UreFD molecules (Fig. 1C). As expected, the R179A/Y183D substitutions disrupted the dimerization of *Hp*UreFD, and each catalytic unit of *H. pylori* urease is interacting with one copy of *Hp*UreD and *Hp*UreF (Fig. 1E). As for the *Kp*UreD/urease complex, *Hp*UreD interacts with the urease (Fig. 1E). The binding sites of *Kp*UreD and *Hp*UreFD on urease are very similar and involve an area of the α domain flanked by the β and γ domains (Fig. 1, F and G). The surface area buried by UreD on *K. pneumoniae* and *H. pylori* ureases were 2225 and 2384 Å^2^, respectively. Residues involved in polar contacts (hydrogen bonds or salt bridges) between UreD and the urease are indicated in Fig. 1 (F and G) and listed in table S1.

### Complex formation induces conformational changes in urease and UreD

**Fig. 2.**
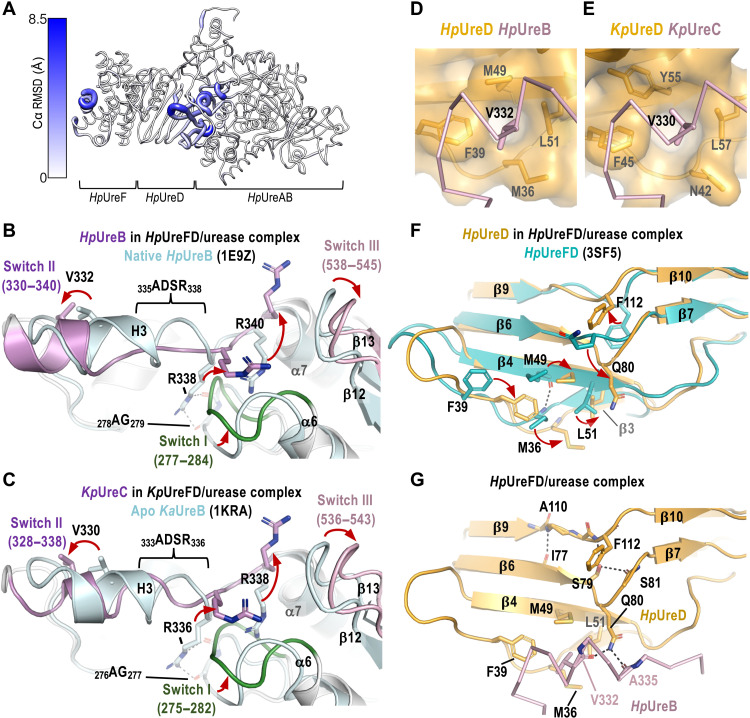
Conformational changes upon the formation of the *Hp*UreFD/urease complex. (**A**) The cryo-EM structure of the *Hp*UreFD/urease (ribbon) is compared to the crystal structures of *Hp*UreFD [Protein Data Bank (PDB): 3SF5] and native urease (*Hp*UreAB) (PDB: 1E9Z). The Cα RMSD values are represented by the thickness of the ribbons and the depth of blue color. (**B**) Formation of the *Hp*UreFD/urease complex induces large conformational changes in three switch regions in *Hp*UreB. Uncoiling of the C-terminal turn of helix H3 (_335_ADSR_338_) causes switches II and III residues to stretch in opposite directions in such a way that Arg^338^ and Arg^340^ flip outward. (**C**) Similar conformational changes are observed when the structure of *Kp*UreD/urease is compared to the structure of *K. aerogenes* urease apoprotein (PDB: 1KRA). (**D**) The stretching motion of helix H3 relocates Val^332^ of *Hp*UreB to dock into a hydrophobic pocket formed by Met^36^, Phe^39^, Met^49^, and Leu^51^ of *Hp*UreD. (**E**) Similar interactions are found in the *Kp*UreD/urease structure. (**F**) *Hp*UreD undergoes structural rearrangement to accommodate the Val^332^ of *Hp*UreB. In particular, the side chain of Phe^112^ of *Hp*UreD swings out from a buried position to become exposed. (**G**) Hydrogen bonds that stabilize the structural rearrangement of *Hp*UreD are depicted in black dotted lines.

The structure of the *Hp*UreFD/urease complex was compared to the crystal structures of *Hp*UreFD (*16*) and *H. pylori* native urease (*4*). In addition to minor structural changes at the C terminus of helix 1 in the dimerization interface of *Hp*UreF, residues with large values of Cα RMSD (root mean square deviation of α carbon) are found in the binding interface between *Hp*UreD and *Hp*UreB (Fig. 2A). The conformational changes upon the formation of *Hp*UreD/urease complex are summarized in Fig. 2 and in movie S1. Binding of UreD induces major conformational changes in three switch regions of *Hp*UreB: (i) the glycine-rich loop (_277_GAGGGHAP_284_) between strand 6 and helix 6, (ii) helix H3 and the loop connecting to helix 7 (residues 330 to 340), and (iii) residues between strand 12 and strand 13 (residues 538 to 545) (Fig. 2B). Upon binding of *Hp*UreFD, residues _335_ADSR_338_ at the C terminus of helix H3 uncoil, causing switches II and III residues to stretch in opposite directions and residues Arg^338^ and Arg^340^ to flip out toward UreD (Fig. 2B). As Arg^338^ is hydrogen bonded to the backbone amide of Ala^278^ and Gly^279^, the conformational change is propagated to the glycine-rich switch I loop (Fig. 2B). When the structure of the *Kp*UreD/urease complex was compared to the crystal structure of *K. aerogenes* urease apoprotein (*3*) and native urease (*21*), similar conformational changes were observed (Fig. 2C and fig. S6).

The formation of the *Hp*UreFD/urease complex also induces major conformational changes in *Hp*UreD (Fig. 2F and movie S1). The stretching motion of helix H3 brings Val^332^ of *Hp*UreB to dock into a hydrophobic pocket of *Hp*UreD formed by Met^36^, Phe^39^, Met^49^, and Leu^51^ (Fig. 2D). This interaction is also conserved in the *Kp*UreD/urease complex, where Val^330^ of *Kp*UreC is interacting with Asn^42^, Phe^45^, Tyr^55^, and Leu^57^ of *Kp*UreD (Fig. 2E). *Hp*UreD undergoes structural rearrangements to accommodate the Val^332^ of *Hp*UreB: Met^36^ moves away from Met^49^ to create a space for binding Val^332^, thereby breaking the backbone hydrogen bond between the two residues; Phe^39^ moves toward Met^36^ to form the hydrophobic pocket with Met^36^, Met^49^, and Leu^51^ (Fig. 2F). On the other hand, Gln^80^ undergoes a swivel motion (Fig. 2F) so that it can form hydrogen bonds to the backbone amides of Val^332^ and Ala^335^ of *Hp*UreB (Fig. 2G). This swivel motion induces structural rearrangement in the regions of strand 6/7 and strand 9/10 in such a way that the side chain of Phe^112^ swings out from a buried to an exposed position (Fig. 2F). The backbone conformations of the loops are stabilized by additional hydrogen bonds involving Ile^77^, Ser^79^, Ser^81^, Ala^110^, and Phe^112^ as indicated in Fig. 2G.

### UreD/urease interaction is important in urease maturation

**Fig. 3.**
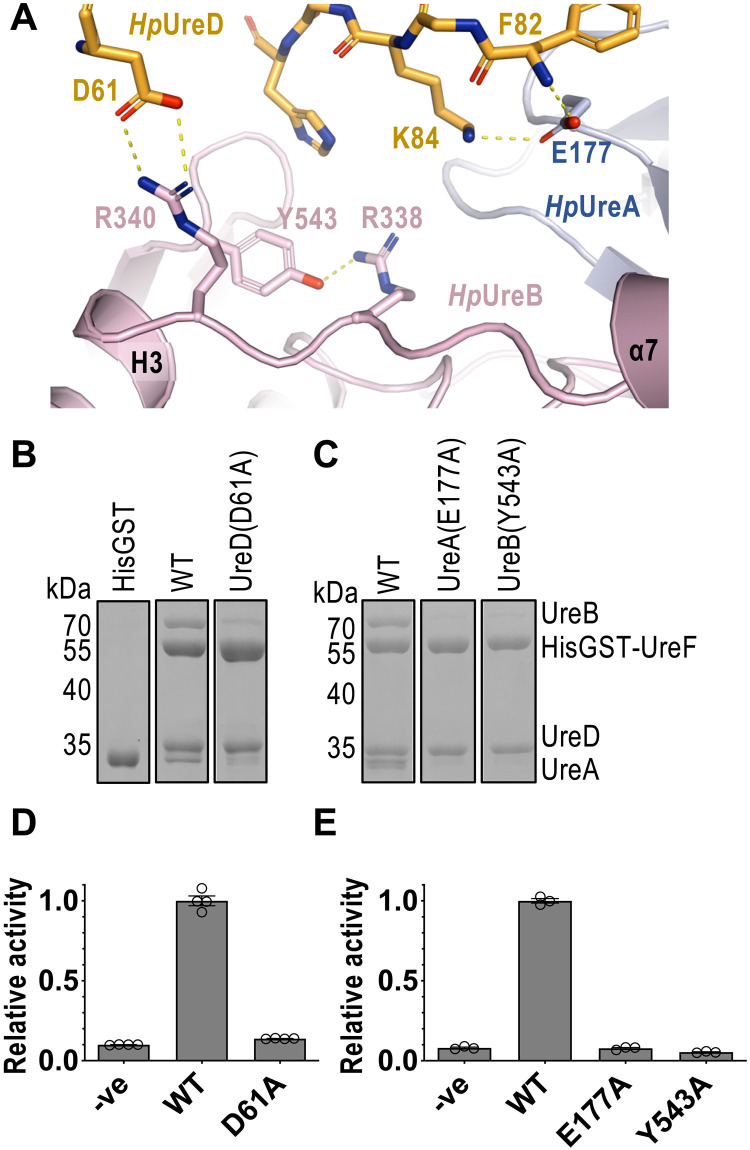
Conserved interactions between UreD and urease are essential to urease maturation. (**A**) Polar interactions involving conserved residues between *Hp*UreD and urease (*Hp*UreAB) are indicated as yellow dashes. (**B** and **C**) The pull-down assay was performed by loading *E. coli* lysates expressing (WT/variant) *Hp*UreAB to GSTrap columns coupled with (WT/variant) HisGST-*Hp*UreFD or HisGST. See fig. S8 for sample preparation. (B) *Hp*UreAB coeluted with the WT HisGST-*Hp*UreFD, but not with the HisGST control. Coelution of *Hp*UreAB with the HisGST-*Hp*UreF/UreD(D61A) variant was greatly reduced. (C) E177A substitution in *Hp*UreA and Y543A substitution in *Hp*UreB essentially abolished coelution of *Hp*UreAB and HisGST-*Hp*UreFD. (**D** and **E**) In vitro urease activation assay. *Hp*UreAB was activated with (WT/variant) *Hp*UreFD and *Hp*UreG in the reaction buffer at 37°C for 30 min. Ni^2+^ was omitted in the reaction buffer in the negative control. Mean relative activity and SEM of at least three measurements were reported. Urease activities of D61A, E177A, and Y543A variants of *Hp*UreD, *Hp*UreA, and *Hp*UreB, respectively, were significantly lower than that of WT [one-way analysis of variance (ANOVA), *P* < 0.0001]. There were no significant differences among variants and the negative control.

The most notable conformational changes observed in urease are the flipping of Arg^338^ and Arg^340^ toward UreD. The conformation of Arg^338^ is stabilized by forming a hydrogen bond to and stacking against Tyr^543^ of *Hp*UreB (Fig. 3A). On the other hand, Arg_340_ of *Hp*UreB forms an intermolecular hydrogen bond with Asp^61^ of *Hp*UreD (Fig. 3A). The interaction between *Hp*UreD and urease is further strengthened by Glu^177^ of *Hp*UreA forming hydrogen bonds with Phe^82^ and Lys^84^ of *Hp*UreD. These hydrogen bonds are also conserved in *Kp*UreD/urease (fig. S7). To test the role of these conserved interactions in urease maturation, we have created a D61A variant of *Hp*UreD, E177A variant of *Hp*UreA, and Y543A variant of *Hp*UreB and tested the interaction between *Hp*UreFD and *Hp*UreAB via a pull-down assay (Fig. 3, B and C). Our results show that wild-type (WT) polyhistidine glutathione *S*-transferase (HisGST) tagged *Hp*UreFD coelutes with *Hp*UreAB, suggesting that *Hp*UreFD interacts with *Hp*UreAB in the pull-down assay (Fig. 3B and fig. S8). In contrast, the interaction between *Hp*UreFD and *Hp*UreAB was greatly reduced by D61A substitution of *Hp*UreD (Fig. 3B), E117A substitution of *Hp*UreA, and Y543A substitution of *Hp*UreB (Fig. 3C). We further show that these substitutions abolish urease activity in an in vitro assay (Fig. 3, D and E). These results suggest that these conserved polar interactions are important in the formation of the *Hp*UreFD/urease complex and in the maturation of urease.

### A tunnel inside the *Hp*UreFD/urease complex facilitates urease maturation

**Fig. 4.**
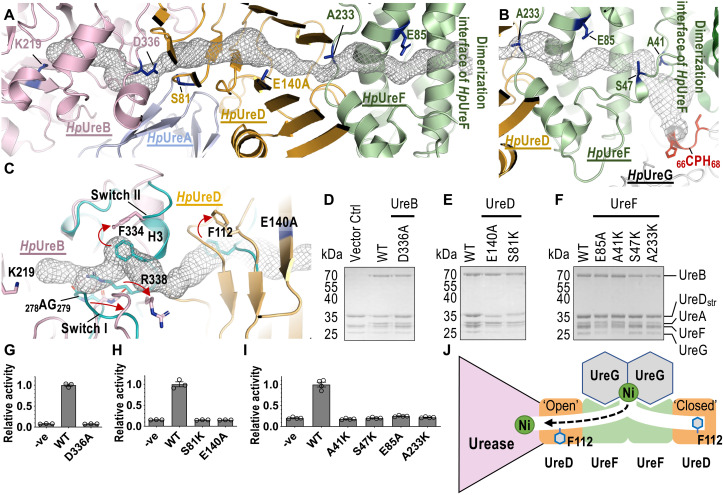
The tunnel inside the *Hp*UreFD/urease complex is important for urease activation. (**A**) A protein tunnel (mesh lines) that connects the active site residue Lys^219^, through *Hp*UreFD, to the dimerization interface of *Hp*UreF is identified in the *Hp*UreFD/UreAB complex. (**B**) A tunnel that passes through the similar region of *Hp*UreF is also identified in the crystal structure of *Hp*UreGFD (PDB: 4HI0). (**C**) The structure of *Hp*UreFD/UreAB complex (orange and pink) is compared to the structures of *Hp*UreGFD (teal; PDB: 4HI0) and *Hp*UreAB (teal; PDB: 1E9Z). In the *Hp*UreFD/UreAB complex, Phe^112^ of *Hp*UreD and switch II residues (Phe^334^ and Arg^338^) of *Hp*UreB relocate to open a tunnel to the urease active site. (**D** to **F**) The pull-down assay was performed by loading *E. coli* lysates expressing (WT/variant) *Hp*UreAB to Strep-Tactin XT resins coupled with (WT/variant) *Hp*UreGFD_str_. See fig. S10 for sample preparation. *Hp*UreAB eluted in all variants tested, but not in the vector control. (**G** to **I**) In vitro urease activation assay. *Hp*UreAB (WT/variant) was activated with (WT/variant) *Hp*UreGFD_str_ in the reaction buffer at 37°C for 30 min. Ni^2+^ was omitted in the reaction buffer in the negative control. Mean relative activity and SEM of at least three measurements were reported. Urease activities of all tunnel-disrupting variants were significantly lowered than that of WT (one-way ANOVA, *P* < 0.0001). There were no significant differences among variants and the negative control. (**J**) A model of how Ni is delivered through a tunnel from UreG to the urease. Phe^112^ of *Hp*UreD serves as a gate residue that relocates from a buried to an exposed position, opening a passage to the urease active site. Upon GTP hydrolysis, the Ni(II) ion is released from UreG, enters the protein tunnel that passes through UreF and UreD, and reaches the urease active site.

We noticed that the formation of the *Hp*UreFD/urease complex opens a tunnel that reaches the active site of urease (Fig. 4A). Tunnel searching was performed using the program CAVER 3.0 (*22*). This 100-Å-long tunnel starts at the active site residue Lys^219^ of urease, exits *Hp*UreB near Asp^336^ of the switch II region, passes through *Hp*UreD between the two layers of β sheets, enters *Hp*UreF near Ala^233^, and reaches the dimerization interface of *Hp*UreF (Fig. 4A). A tunnel was also identified in the *Kp*UreD/urease complex that passes through similar regions in *Kp*UreC and *Kp*UreD (fig. S9). In the crystal structure of the *Hp*UreGFD complex (*17*), a tunnel that passes through a similar region of *Hp*UreF was identified (Fig. 4B) (*23*–*25*). Together, these observations suggest that the tunnel inside the *Hp*UreFD/urease complex connects the active site of urease (Fig. 4A) to the CPH Ni binding motif of UreG (Fig. 4B).

Conformational changes in the *Hp*UreB/UreD interface are instrumental in the opening of the tunnel that reaches the urease active site (Fig. 4C and movie S2). The active site residue Lys^219^, which is carbamylated and binds Ni(II) ions in mature urease, is completely buried inside urease. The access to the active site is blocked by the glycine-rich switch I loop and switch II residues (e.g., Phe^334^ and Arg^338^) of *Hp*UreB (Fig. 4C). These residues relocate upon the formation of the *Hp*UreFD/urease complex, making room to create a tunnel that reaches the active site of urease. On the other hand, the tunnel in the *Hp*UreD is blocked by Phe^112^ before the formation of the *Hp*UreFD/urease complex (Fig. 4C). As described above (Fig. 2F), the UreD/urease interaction causes Phe^112^ to flip to an exposed position and thereby open a passage that connects the tunnel between *Hp*UreB and *Hp*UreD (Fig. 4C).

To test whether the tunnel is essential for Ni delivery to the urease, we introduced tunnel-disrupting substitutions to residues along the tunnel. We first introduced charge-to-alanine substitutions in acidic residues buried inside the tunnel (i.e., D336A of *Hp*UreB, E140A of *Hp*UreD, and E85A of *Hp*UreF). We hypothesized that these conserved acidic residues (refer to the alignment in figs. S3 and S5) are important in stabilizing the positively charged Ni(II) ions inside the tunnel. The second strategy was to introduce lysine substitutions to small amino acid residues along the tunnel (i.e., S81K of *Hp*UreD and A41K, S47K, and A233K of *Hp*UreF) because our modeling suggested that the flexible lysine side chain can block the tunnel without affecting the proper folding of *Hp*UreFD.

We first coexpressed *Hp*UreD_str_ with *Hp*UreF and *Hp*UreG in *E. coli* and purified the resulting *Hp*UreGFD_str_ complex by Strep-Tactin affinity chromatography (fig. S10A). We show that the tunnel-disrupting substitutions in *Hp*UreD (S81K and E140A) and *Hp*UreF (A41K, S47K, E85A, and A233K) did not affect the formation of the *Hp*UreGFD_str_ complex (fig. S10, C and E), and the purified *Hp*UreGFD_str_ interacted with WT *Hp*UreAB in a pull-down assay (Fig. 4D). The interaction was not affected by tunnel-disrupting substitutions in *Hp*UreB (D336A; Fig. 4D), *Hp*UreD (S81K and E140A; Fig. 4E), and *Hp*UreF (A41K, S47K, E85A, and A233K; Fig. 4F). These observations suggest that the tunnel-disrupting substitutions do not disrupt formation of the activation complex between the urease and its accessory proteins. On the other hand, all these tunnel-disrupting substitutions abolished urease activity (Fig. 4, G to I). As shown in Fig. 4 (A and B), these tunnel-disrupting substitutions are distributed along the 100-Å-long tunnel—D336A and S81K are located at the *Hp*UreB/UreD interface, E140A is located inside *Hp*UreD, A233K is located at the *Hp*UreD/UreF interface, E85A is located inside *Hp*UreF, and A41K and S47K are located at the dimeric interface of *Hp*UreF. Together, our results suggest that Ni(II) ions are delivered along this tunnel, passing through *Hp*UreF and *Hp*UreD, to reach the active site of urease.

To test whether urease maturation requires dimerization of UreFD, we performed the urease activation assay in two-chamber dialyzers using the strategy described previously (fig. S11) (*15*). In this assay, Ni-bound *Hp*UreG provides the sole source of Ni for urease activation. Our results showed that urease was activated when Ni-bound *Hp*UreG was mixed with WT *Hp*UreFD and *Hp*UreAB in the same chamber (fig. S11A). On the other hand, when Ni-bound *Hp*UreG was separated from *Hp*UreFD and *Hp*UreAB by a dialysis membrane, urease activation was abolished (fig. S11D). Substitutions of R179A/Y183D in *Hp*UreF, which breaks dimerization of *Hp*UreFD, abolished urease activation in vitro (fig. S11B). We have previously showed that the R179A/Y183D substitutions abolished the formation of *Hp*UreGFD complex and inhibited urease maturation in vivo (*17*). Together, our results suggest that Ni delivery requires *Hp*UreG to interact with the dimeric *Hp*UreFD in complex with urease.

## DISCUSSION

To avoid cytotoxicity, ions such as copper and Ni are tightly regulated to subnanomolar concentrations (corresponding to less than one ion per bacterial cell) (*26*). To activate the urease, the enzyme cannot just pick up free Ni(II) ions from the cytoplasm. Instead, the Ni(II) ions are acquired by metallochaperones such as UreE and UreG and are delivered to the active site of urease within specific protein complexes so that the toxic metal ions do not diffuse into the cytoplasm. After receiving its Ni(II) ions from the UreE_2_G_2_ complex (*15*, *18*), Ni-bound UreG activates urease by forming a UreGFD/urease activation complex (*17*, *20*). When Ni-bound UreG was separated from the UreFD/urease complex by a dialysis membrane in a two-chamber dialyzer, the urease activation was abolished (*15*). This observation suggests that direct protein-protein interaction between UreG and UreFD/urease is essential for Ni delivery from UreG to the urease (*15*). The dimerization-deficient variant of UreF(R179A/Y183D) failed to form the UreGFD complex and to activate urease in vitro (fig. S11) and in vivo (*17*), suggesting that UreG delivers its Ni through interaction with the UreFD dimer and the urease. Ni-bound UreG is likely interacting with UreF in the activation complex, as substitutions that break UreG/UreF interaction also abolish urease maturation (*16*, *27*).

We have modeled the structure of the *Hp*UreGFD/urease activation complex based on the cryo-EM structure of *Hp*UreFD/urease (this study) and the crystal structure of the guanosine diphosphate (GDP)–bound *Hp*UreGFD complex (fig. S12) (*17*) to propose a model of how Ni is delivered from UreG to urease (Fig. 4J). A tunnel can be identified in the activation complex that connects the active site of urease to the Ni binding site of UreG (fig. S12). In the GTP-bound state of UreG, the Ni(II) ion is bound at the dimer interface by Cys^66^ and His^68^ of the UreG-conserved CPH motif (*15*). UreG is modeled in the GDP-bound state and may represent the structure of the activation complex immediately after GTP hydrolysis, which disrupts the Ni-binding square-planar coordination ligands (fig. S12). Our mutagenesis and biochemical studies suggest that Ni released from UreG can pass through UreF to UreD to reach the urease buried active site via this tunnel (Fig. 4J).

A protein tunnel can also be defined with the *Hp*UreGFD complex and predicted in *K. aerogenes* UreD (*17*, *23*–*25*, *28*). The tunnel in *Hp*UreGFD follows a similar path to that identified in the *Hp*UreFD/urease complex until it reaches the region near Glu^140^ of *Hp*UreD. Molecular dynamics simulations suggest that hydrated Ni(II) ions can pass through this tunnel from the Ni-binding CPH motif of UreG to Glu^140^ of UreD inside the *Hp*UreGFD complex (*25*). Comparing the structures of the *Hp*UreFD/urease and *Hp*UreGFD complexes reveal that Phe^112^ of *Hp*UreD can serve as a gate residue that blocks the tunnel in the *Hp*UreGFD complex (Fig. 4C).

The *Hp*UreD gate residue only opens to allow access to the urease active site following the conformational changes in *Hp*UreD and *Hp*UreB induced by formation of the activation complex (Fig. 4C and movie S2). In particular, the gate residue Phe^112^ relocates from a buried position to an exposed position, thus “opening” the tunnel to allow passage of Ni(II) ions to the urease active site (Fig. 4J). On the other hand, Phe^112^ of *Hp*UreD on the other side of the activation complex, away from the urease, is expected to adopt the buried position that “closes” the tunnel (Fig. 4J). The closure of the tunnel ensures the Ni(II) ion does not diffuse into the cytoplasm at the other end of the complex. Our model also explains how GTP hydrolysis thermodynamically drives the Ni delivery to the urease—because GTP hydrolysis disrupts the Ni binding site at UreG, the released Ni(II) ion is trapped inside the activation complex until it reaches the active site of urease, where it can form more stable interactions.

The activation complex should dissociate after urease is activated. In native *H. pylori* urease, the Ni(II) ions are coordinated by carbamylated Lys^219^, His^136^, His^138^, His^274^, and Asp^320^ (fig. S13A). Structural comparison reveals that Ni binding to the active site relocates His^274^ in a position to form a hydrogen bond to Gly^280^ of switch II (fig. S13A). This interaction promotes conformational changes in switches I and II such that Arg^338^ flips back toward the active site to form hydrogen bonds to Ala^278^ and Gly^279^, inducing dissociation of UreD from the urease. Similar conformational changes are also conserved in *Klebsiella* urease (fig. S13B). These observations suggest that Ni binding to the active site provides additional interactions that promote dissociation of the activation complex.

In summary, this study shows how cells solve the problem of delivering a toxic metal ion to an essential enzyme by opening a 100-Å-long tunnel within the activation complex so that the toxic Ni(II) ion is delivered through the tunnel to the urease active site. The urease maturation pathway thus provides a paradigm on trafficking of toxic metal ions in cells. The delivery of Ni(II) ions along the urease maturation pathway always occurs within protein complexes so that the toxic metal ions cannot escape into the cytoplasm. In addition to academic curiosity, because *H. pylori* requires active urease to survive in the acidic environment of human stomach (*2*), a better understanding of the mechanism of the urease maturation pathway could provide insights into the development of treatment for *H. pylori* infection (*29*).

## MATERIALS AND METHODS

### Plasmids construction and mutagenesis

Figure S14 summarizes the constructs used in this study. The construction of pHpA2H and introduction of R179A/Y178D substitutions to *Hp*UreF were described previously (*14*, *15*). To create pHpA2H_str_-UreF(R179A/Y183D)UreD(E140A), E140A substitution and a C-terminal Strep-tag II (WSHPQFEK) were added to *Hp*UreD (encoded by *ureH*). To create the plasmid pKpUreD_str_ABC, the *K. pneumoniae* gene cluster *ureDABC* was cloned between the Nde I and Xho I sites of the pRSF-Duet1 vector (Novagen) with a Strep-tag II fused to the N terminus of *Kp*UreD. The construction of plasmids pHisSUMO-HpUreG, pHisGST-HpUreF, pHpUreH, and pHpUreAB (encoding HisSUMO-*Hp*UreG, HisGST-*Hp*UreF, *Hp*UreD, and *Hp*UreAB, respectively) used in this work was described previously (*16*, *17*). To create the plasmid pHpUreGFD_str_, the *H. pylori* gene cluster *ureFGH* was cloned between the Nde I and Eco RI sites of an in-house pRSETA (Invitrogen) vector with a Strep-tag II fused to the C terminus of *Hp*UreD. To create the plasmid pHisGST, the coding sequence of the GST was cloned between the Eco R1 and Pas I sites of the pET-Duet1 vector (Novagen) with an N-terminal polyhistidine tag. Variants of *Hp*UreA, *Hp*UreD, and *Hp*UreF were generated using the Q5 mutagenesis kit (New England Biolabs) or the QuikChange II site-directed mutagenesis kit (Agilent) following the protocols from the manufacturers. Variants of *Hp*UreB were generated by overlap extension polymerase chain reaction (*30*). Primers used for site-directed mutagenesis are listed in the table S2.

### Structure determination of the *Hp*UreFD/urease and *Kp*UreD/urease complexes

#### Protein sample preparation

*E. coli* BL21 was transformed with the expression plasmids of pHpA2H_str_-UreF(R179A/Y183D)UreD(E140A) and pKpUreD_str_ABC, cultured in LB at 37°C, and induced overnight with 0.4 mM isopropyl β-d-thiogalactopyranoside (IPTG) when optical density at 600 nm (OD_600_) reached 0.5. The harvested cells were resuspended in the Strep-binding buffer [50 mM Hepes, 200 mM NaCl, and 0.5 mM TCEP (pH 7.5)] and lysed using EMULSIFLEX-C5. The soluble lysate was loaded onto a Strep-Tactin XT column (IBA Lifesciences), washed extensively with the Strep-binding buffer, and eluted with 50 mM d-biotin in the Strep-binding buffer. The *Hp*UreFD/urease and *Kp*UreD/urease complex were further purified by size exclusion chromatography using a Superose-6 10/300 column (GE HealthCare) pre-equilibrated with the Strep-binding buffer. Quantifoil R1.2/1.3 copper grids (200 mesh) with holey carbon foil were glow discharged for 20 s at 15 mA. Four microliters of *Kp*UreD/urease (0.2 mg/ml) or *Hp*UreFD/urease complex (0.5 mg/ml) was applied to the grids. Grids were plunge-frozen in liquid ethane using the Vitrobot Mark IV (Thermo Fisher Scientific) maintained at 100% humidity at 4°C, with blot time of 3 s and blot force of 0.

#### Collection and processing of cryo-EM data

For the structure determination of the *Hp*UreFD/urease complex, 1953 movies were collected using a K2 detector on a Titan G3 microscope at a calibrated pixel size of 0.822 Å. Each movie was imaged with a total dose of 48e^−^/Å^2^. Preprocessing was streamed using SIMPLE3.0 (*31*) including patched motion correction (5 × 5 patches), patched contrast transfer function (CTF) correction (5 × 5 patches), and auto picking using a template derived from two-dimensional (2D) averages generated from 200 handpicked particles from a preliminary run. A total of 166,893 particles were picked. Two rounds of 2D classification led to a set of 132,431 particles, and an initial model was generated from the 2D class averages using SIMPLE3.1 with tetrahedral symmetry imposed. The particles were exported to RELION3.0 (*32*), and 3D classification into three classes led to identification of a subset of 68,516 particles with higher occupancy for the UreFD components. Further rounds of 3D refinement, CTF refinement, and particle polishing led to a volume of 2.3-Å resolution [as assessed by gold standard Fourier shell correlation (FSC) = 0.143 criterion].

For the structure determination of the *Kp*UreD/urease complex, 3149 movies were collected using a K2 detector on a Titan G3 microscope at a calibrated pixel size of 0.822 Å. Each movie was imaged with a total dose of 48e^−^/Å^2^. Preprocessing was streamed using SIMPLE3.0 including patched motion correction (5 × 5 patches), patched CTF correction (5 × 5 patches), and auto picking using a template derived from 2D averages generated from ~200 handpicked particles. A total of 567,251 particles were picked. Rounds of 2D classification led to a set of 476,622 particles, and an initial model was generated in SIMPLE3.0 from the 2D class averages. 3D classification within RELION3.0 into six classes led to identification of a subset of 111,242 particles with higher occupancy of the UreD component on all three vertices. Additional rounds of 2D classification pruned this subset to 89,857 particles before further rounds of 3D refinement with C3 symmetry imposed, CTF refinement, and particle polishing, leading to a volume of 2.7-Å resolution (as assessed by gold standard FSC = 0.143 criterion).

#### Model building and refinement

Initial models for the *Kp*UreD/urease complex were derived from the crystal structure of *K. aerogenes* urease apoprotein [Protein Data Bank (PDB): 1KRA] (*3*), and a *Kp*UreD model was generated by homology modeling using the program MODELLER implemented in UCSF CHIMERA (*33*). Initial models for the *Hp*UreFD/urease complex were derived from the crystal structure of *H. pylori* native urease (PDB: 1E9Z) (*4*) and the structure of *Hp*UreFD from the *Hp*UreGFD complex (PDB: 4HI0) (*17*). These initial models were fitted into the cryo-EM maps of the *Kp*UreD/urease and the *Hp*UreFD/urease complexes using the program UCSF CHIMERA (*34*). Models were built interactively using the program COOT (*35*) and refined using the program PHENIX.REAL_SPACE_REFINE (*36*).

### Structure analysis

Tunnel searching was performed by the program CAVER 3.0 (*22*) implemented in the program PyMOL (https://pymol.org) using the default setting of a 0.9-Å probe radius. The structures of the *Hp*UreFD/urease and *Kp*UreD/urease complexes were superimposed to the native structure of *H. pylori* and *K. aerogenes* ureases (PDB: 1E9Z and 1FWJ) to identify the location of the Ni binding sites, which serve as the starting point of the tunnel search. The movies S1 and S2 were created using the program PyMOL to morph the structures of *Hp*UreAB (PDB: 1E9Z) and *Hp*UreFD (PDB: 3SF5) into the structure of *Hp*UreFD/UreAB (this study).

### Pull-down assay for testing interactions between *Hp*UreFD and *H. pylor* urease

#### Protein sample preparation

To prepare samples of WT and variants of HisGST-*Hp*UreFD, *E. coli* Rosetta or C41 (DE3) was cotransformed with pHisGST-HpUreF and pHpUreH (WT/variant), cultured in King Broth or Terrific Broth with appropriate antibiotics [ampicillin (100 μg/ml), kanamycin (50 μg/ml), and chloramphenicol (25 μg/ml)], induced overnight with 0.4 mM IPTG when OD_600_ reached 0.6 to 0.8 at 25°C. The harvested cells were resuspended in the assay buffer [20 mM Hepes, 200 mM NaCl, and 1 mM tris(2-carboxyethyl)phosphine hydrochloride (TCEP-HCl) (pH 7.5)] supplemented with 1 mM phenylmethylsulfonyl fluoride or cOmplete ULTRA Tablets protease inhibitor cocktail (0.01 g/ml; Roche). After lysis by sonication, the cell lysates were loaded onto a 5-ml HisTrap Ni-chelating column (GE HealthCare) pre-equilibrated with 45 mM imidazole in the assay buffer. HisGST-*Hp*UreFD was eluted with 300 mM imidazole in the assay buffer (fig. S8, A and C). The negative control samples of HisGST were obtained using the same protocols by transforming *E. coli* C41 (DE3) with the expression plasmid of pHisGST (fig. S8A).

To prepare cell lysates expressing WT and variants of *Hp*UreAB, *E. coli* Rosetta (DE3) was transformed with pHpUreAB (WT/variants), cultured in King Broth or Terrific Broth with appropriate antibiotics [kanamycin (50 μg/ml) and chloramphenicol (25 μg/ml), induced overnight with 0.4 mM IPTG when OD_600_ reached 0.6 to 0.8 at 25°C. Each gram of cell pellet was resuspended in 10 ml of the assay buffer and lysed by sonication. After centrifuging at 20,000*g* for 30 min, the soluble lysates were collected and filtered using 0.22-μm filters. The concentrations of WT and variants of *Hp*UreAB in the lysates were normalized according to Coomassie Blue staining (fig. S8D).

#### Pull-down assay

Five milliliters of protein samples of (WT/variant) HisGST-HpUreFD or HisGST at 20 μM was loaded onto 5-ml GSTrap FF columns (GE HealthCare) and incubated at 37°C for 30 min. After the columns were washed with 30 ml of assay buffer, 5 ml of lysates of *E. coli* expressing (WT/variant) *Hp*UreAB was added to the columns and incubated at 37°C for another 30 min. The columns were washed with 30 ml of assay buffer, eluted with 10 mM GSH in the assay buffer, analyzed by SDS–polyacrylamide gel electrophoresis (SDS-PAGE) and stained with Coomassie Blue (Fig. 3, B and C).

### Pull-down assay for testing interactions between *Hp*UreGFD_str_ and *H. pylori* urease

#### Protein sample preparation

To purify protein samples of WT or variants of *Hp*UreGFD_str_, *E. coli* BL21 (DE3) pLysS was transformed with pHpUreGFD_str_ (WT/variants), cultured in Terrific Broth with appropriate antibiotics [ampicillin (100 μg/ml) and chloramphenicol (25 μg/ml), induced overnight with 0.4 mM IPTG when OD_600_ reached 0.6 to 0.8 at 25°C. One gram of harvested cells was resuspended in 10 ml of Strep-binding buffer [50 mM Hepes, 200 mM NaCl, and 1 mM TCEP-HCl (pH 7.5)] supplemented with 0.1 g of cOmplete ULTRA Tablets protease inhibitor cocktail (Roche). After lysis by sonication, the cell lysate was collected by centrifugation at 20,000*g* for 40 min and filtered using 0.22-μm filters. A total of 0.5 mM GDP and 1 mM MgSO_4_ were added to the cell lysate, which was then loaded onto 0.5-ml Strep-Tactin XT resins (IBA Lifesciences) pre-equilibrated with the Strep-binding buffer in a spin column. After washing with 8 ml of the Strep-binding buffer, the final trace amount of the buffer was removed by centrifugation at 100*g* for 10 s. Bound proteins were eluted with 2.4 ml of Strep-elution buffer [50 mM d-biotin, 50 mM Hepes, 200 mM NaCl, and 1 mM TCEP-HCl (pH 7.5)] (fig. S10, A, C, and E).

To prepare cell lysates expressing WT and variant of *Hp*UreAB, *E. coli* Rosetta (DE3) was transformed with pHpUreAB (WT/variants), cultured in King Broth or Terrific Broth with appropriate antibiotics [kanamycin (50 μg/ml) and chloramphenicol (25 μg/ml)], induced overnight with 0.4 mM IPTG when OD_600_ reached 0.6 to 0.8 at 25°C. To prepare cell lysates of the vector control, *E. coli* Rosetta (DE3) was transformed with the empty vector of pRSF-Duet1 (Novagen) instead. Each gram of cell pellet was resuspended in 10 ml of the Strep-binding buffer and lysed by sonication. After centrifuging at 20,000g for 40 min, the soluble lysates were collected and filtered using 0.22-μm filters. The concentrations of WT and variants of *Hp*UreAB in the lysates were normalized according to Coomassie Blue staining (fig. S10B). The cell lysate was supplemented with 1 mM GTP and 1 mM MgSO_4_.

#### Pull-down assay

A total of 0.1 ml of 20 μM *Hp*UreGFD_str_ (WT/variants) was loaded onto 0.1-ml Strep-Tactin XT resins (IBA Lifesciences) pre-equilibrated with the Strep-binding buffer in a spin column. After washing the resins with 8 bed volumes of the assay buffer, 100 μl of lysate of *Hp*UreAB (WT/variants) was loaded onto the resins. After incubation at 37°C for 15 min, the resins were washed with 13 bed volumes of the assay buffer. The last trace of buffer was removed by centrifugation at 100*g* for 10 s. Bound proteins were eluted by 0.25 ml of the Strep-elution buffer, analyzed by SDS-PAGE, and stained with Coomassie Blue (Fig. 4, D to F).

### In vitro urease activation assay

#### Protein sample preparation

*Hp*UreG and *Hp*UreFD were purified as described previously (*15*). The *Hp*UreGFD_str_ complex was purified using Strep-Tactin affinity chromatography as described above. To purify *H. pylori* urease apoprotein (*Hp*UreAB), *E. coli* Rosetta (DE3) was transformed with pHpUreAB (WT/variants) cultured in King Broth or Terrific Broth with appropriate antibiotics [kanamycin (50 μg/ml) and chloramphenicol (25 μg/ml), induced overnight with 0.4 mM IPTG when OD_600_ reached 0.6 to 0.8 at 25°C. The cell pellet was resuspended in buffer A [20 mM tris and 1 mM TCEP (pH 8.0)]. After sonication, cell lysate was loaded onto a HiTrap Q HP column (GE HealthCare) pre-equilibrated with buffer A. The column was washed with buffer A and eluted with a 100-ml linear gradient of 0 to 500 mM NaCl in buffer A. Fractions corresponded to ~200 to 325 mM NaCl were collected and concentrated to OD_280_ of ~24 to 27. Two hundred fifty microliters of the sample was loaded onto a Superdex 200 Increase 10/300 column (GE HeathCare) pre-equilibrated with the assay buffer [20 mM Hepes, 200 mM NaCl, and 1 mM TCEP-HCl (pH 7.5)]. Fractions that correspond to the *Hp*UreAB dodecamer (~10.5 ml) were collected, concentrated, and loaded onto a Superose 6 Increase 10/300 column (GE HeathCare) pre-equilibrated with the assay buffer. Purified *Hp*UreAB dodecamer was eluted at ~13.5 ml.

#### Urease activation assay

For the urease activation assay in Fig. 3, 40 μM *Hp*UreG and 40 μM *Hp*UreFD (WT/variants) were mixed with 10 μM *Hp*UreAB (WT/variants) in the reaction buffer [20 μM NiSO_4_, 2 mM MgSO_4_, 0.3 mM GTP, 20 mM Hepes, 200 mM NaCl, and 1 mM TCEP (pH 7.5)]. For the urease activation assay in Fig. 4, 40 μM *Hp*UreGFD_str_ (WT/variants) were mixed with 10 μM *Hp*UreAB (WT/variants) in the reaction buffer instead. NiSO_4_ was omitted in the negative control. KHCO_3_ (10 mM) was added to stimulate the GTP hydrolysis of *Hp*UreG (*17*, *18*) and initiate the urease activation. The reaction mixture was incubated at 37°C for 30 min. Activation of urease was measured as described (*15*). For the urease activation assay in fig. S11, 40 μM Ni-bound or apo-*Hp*UreG, (WT/variant) *Hp*UreFD, and 10 μM *Hp*UreAB were added to either side of a two-chamber dialyzer (Bioprobes Ltd.) separated by a dialysis membrane with a molecular weight cutoff of 6 to 8 kDa (Spectrum Labs). The buffer in both chambers contained 2 mM MgSO_4_, 1 mM GTP, 20 mM Hepes (pH 7.5), 200 mM NaCl, and 1 mM TCEP. After equilibration at 4°C for 16 hours, 10 mM KHCO_3_ was added to both chambers to activate the GTP hydrolysis required for urease activation. The chambers were then incubated at 37°C for 1 hour, and the urease activity was measured as described (*15*). Urease activities were normalized to those measured for using WT proteins and were analyzed with one-way analysis of variance (ANOVA) followed by the Tukey post hoc test using the program Prism (GraphPad). In our hand, specific activity of activated urease ranged from 27 to 181 μmol min^−1^ mg^−1^ (table S3). In comparison, the specific activity of native urease purified from *H. pylori* was 1693 μmol min^−1^ mg^−1^ (*37*).
