# Supplementary material for: Delivering a toxic metal to the active site of urease
Source: Sci Adv. 2023 Apr 21;9(16):eadf7790. doi: 10.1126/sciadv.adf7790 (PMC10121161; doi:10.1126/sciadv.adf7790)
Supplement: Supplementary file 1 — Figs. S1 to S14 Tables S1 to S3 Legends for movies S1 and S2 [file sciadv.adf7790_sm.pdf]

Science Advances
AAAS

Supplementary Materials for

Delivering a toxic metal to the active site of urease

Yap Shing Nim *et al.*

Corresponding author: Susan M. Lea, susan.lea@nih.gov; Kam-Bo Wong, kbwong@cuhk.edu.hk

Sci. Adv. **9**, eadf7790 (2023)
DOI: 10.1126/sciadv.adf7790

The PDF file includes:

Figs. S1 to S14
Tables S1 to S3
Legends for movies S1 and S2

Other Supplementary Material for this manuscript includes the following:

Movies S1 and S2

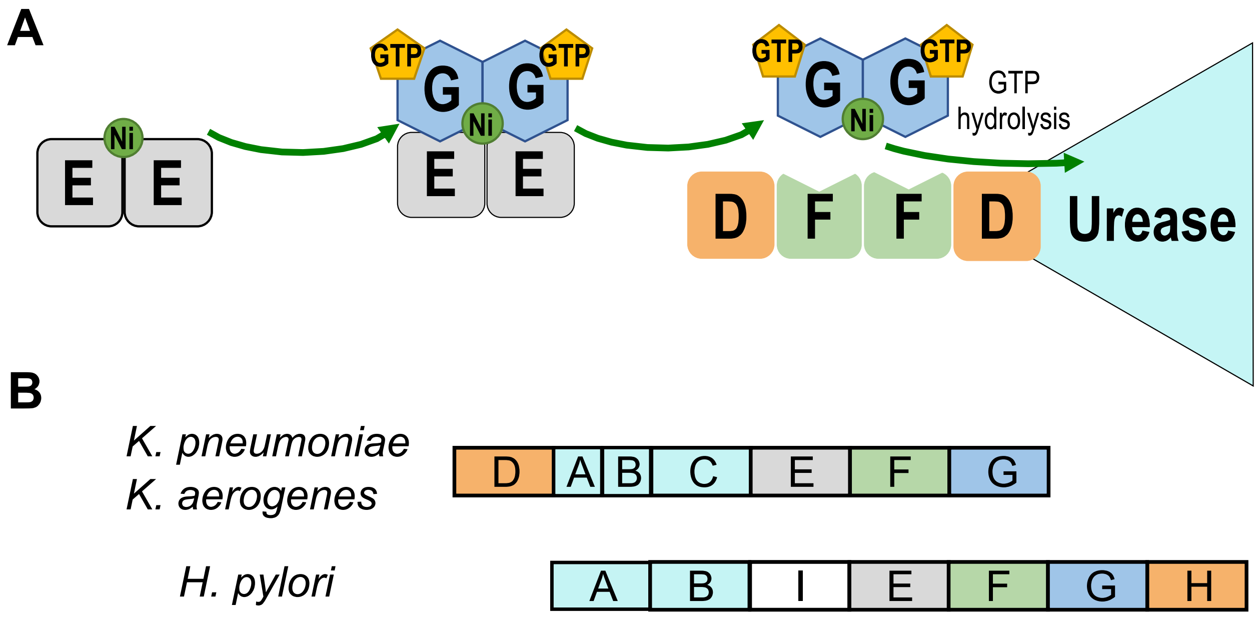

Fig. S1. The urease maturation pathway. (A) Nickel ions are delivered along the urease maturation pathway within the protein complexes so that the toxic metal ions do not leak to the cytoplasm. UreE delivers its nickel ion to UreG via the formation of a $UreE_2G_2$ complex. After receiving its nickel ion, UreG forms an activation complex with UreFD and urease. Upon GTP hydrolysis, nickel is delivered from UreG to urease with the help of UreFD. (B) Urease and urease accessory proteins are encoded in the urease operon in bacteria. In *Klebsiella pneumoniae* and *K. aerogenes, ureA*, *ureB* and *ureC* genes encode the γ, β, α domains of urease, respectively. In *Helicobacter pylori*, *ureA* encodes a fusion protein containing the γ and β domains, while *ureB* encodes the α domain of urease. *ureH* is an ortholog of *ureD.* In this paper, we use UreD to denote the protein products of *ureD* and *ureH.*

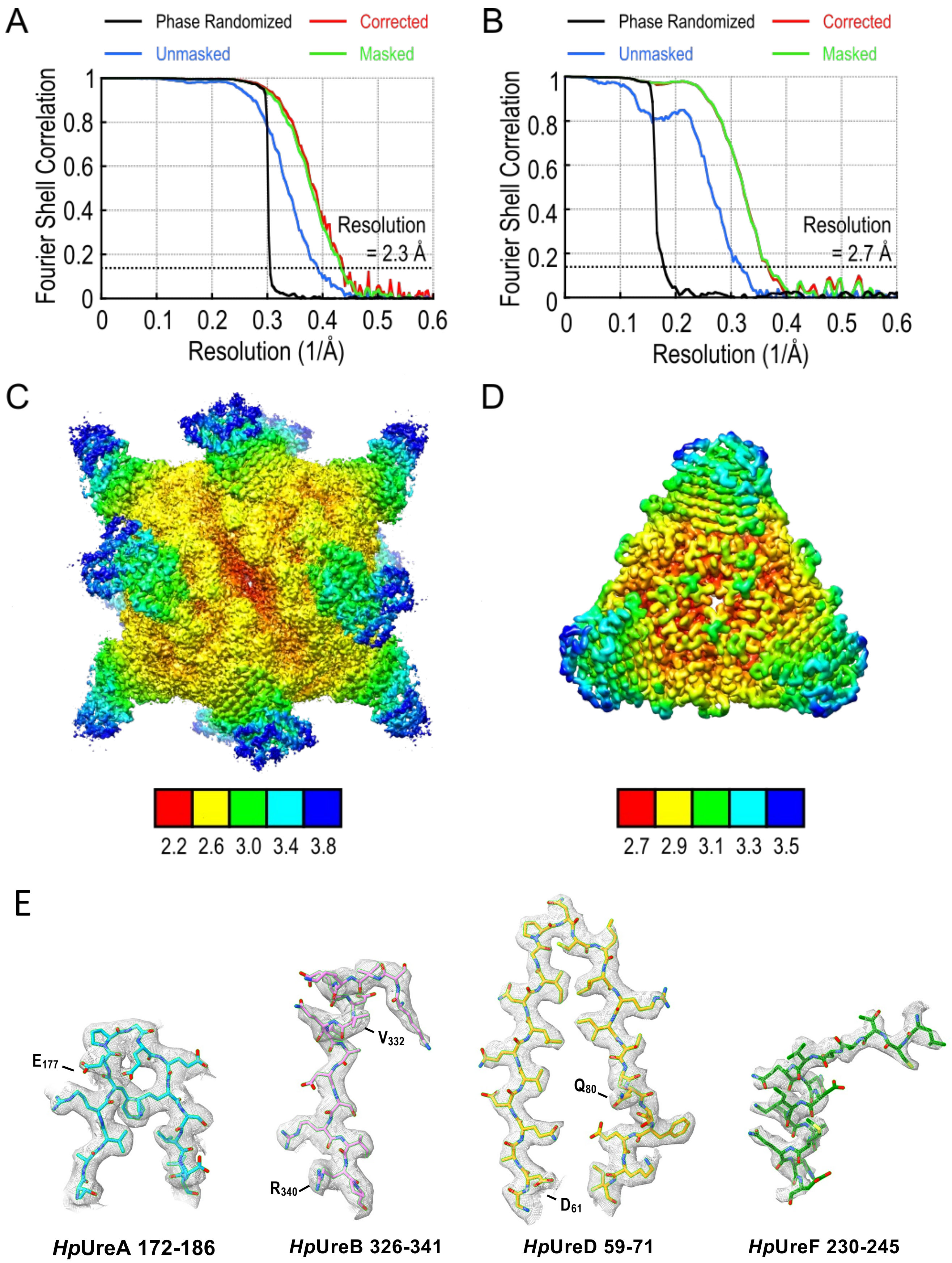

Fig. S2. Quality of the cryo-EM maps of *Hp*UreFD/urease and *Kp*UreD/urease. (A-B) Fourier shell correlation curves of (A) *Hp*UreFD/urease and (B) *Kp*UreD/urease. The final resolution was determined at the gold standard cutoff of 0.143. (C-D) Local resolution of (C) *Hp*UreFD/urease and (D) *Kp*UreD/urease. (E) Examples of the density map of *Hp*UreA, *Hp*UreB, *Hp*UreD and *Hp*UreF contoured at 2.5σ, 2.5σ, 2.0σ and 1.5σ, respectively. Residues that are involved in *Hp*UreD/urease interactions are indicated.

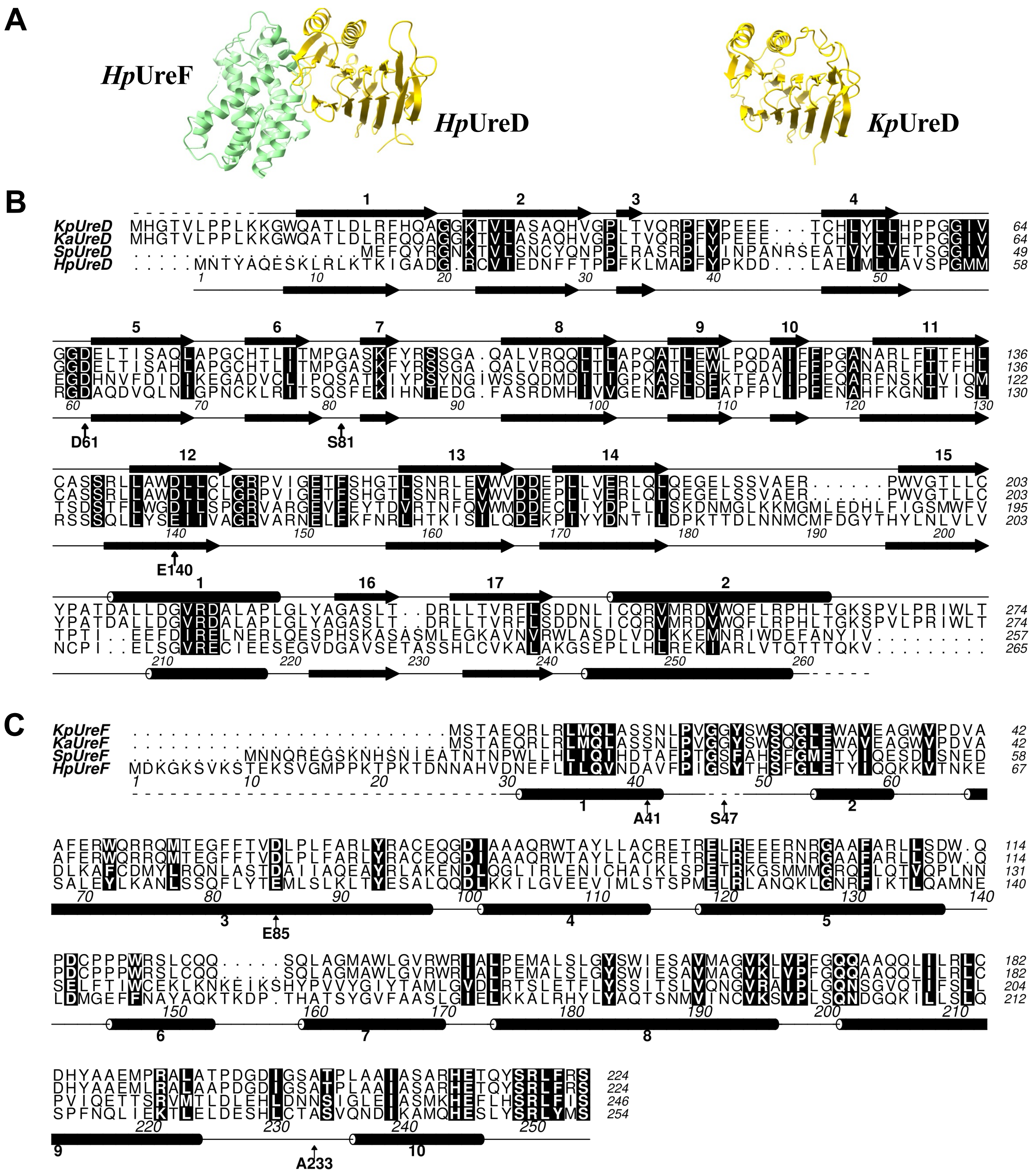

Fig. S3. Structures of UreD and UreF in *Hp*UreFD/urease and *Kp*UreD/urease complexes. (A) Cartoon representation of *Hp*UreFD (left) and *Kp*UreD (right). (B-C) Sequence alignment of UreD and UreF from *K. pneumoniae (Kp)*, *K. aerogenes (Ka)*, *Sporosarcina pasteurii (Sp)* and *H. pylori (Hp)*. Secondary structure elements of the *Kp* and *Hp* proteins are indicated above and below the alignment, respectively. Residues that could not be modelled (1-10 of *Kp*UreD, 261-265 of *Hp*UreD, 1-29 and 46-48 of *Hp*UreF) are indicated by dotted lines. The residue numbers of the *H. pylori* sequences are labelled below the alignment. The residues chosen for mutagenesis studies are indicated by arrows.

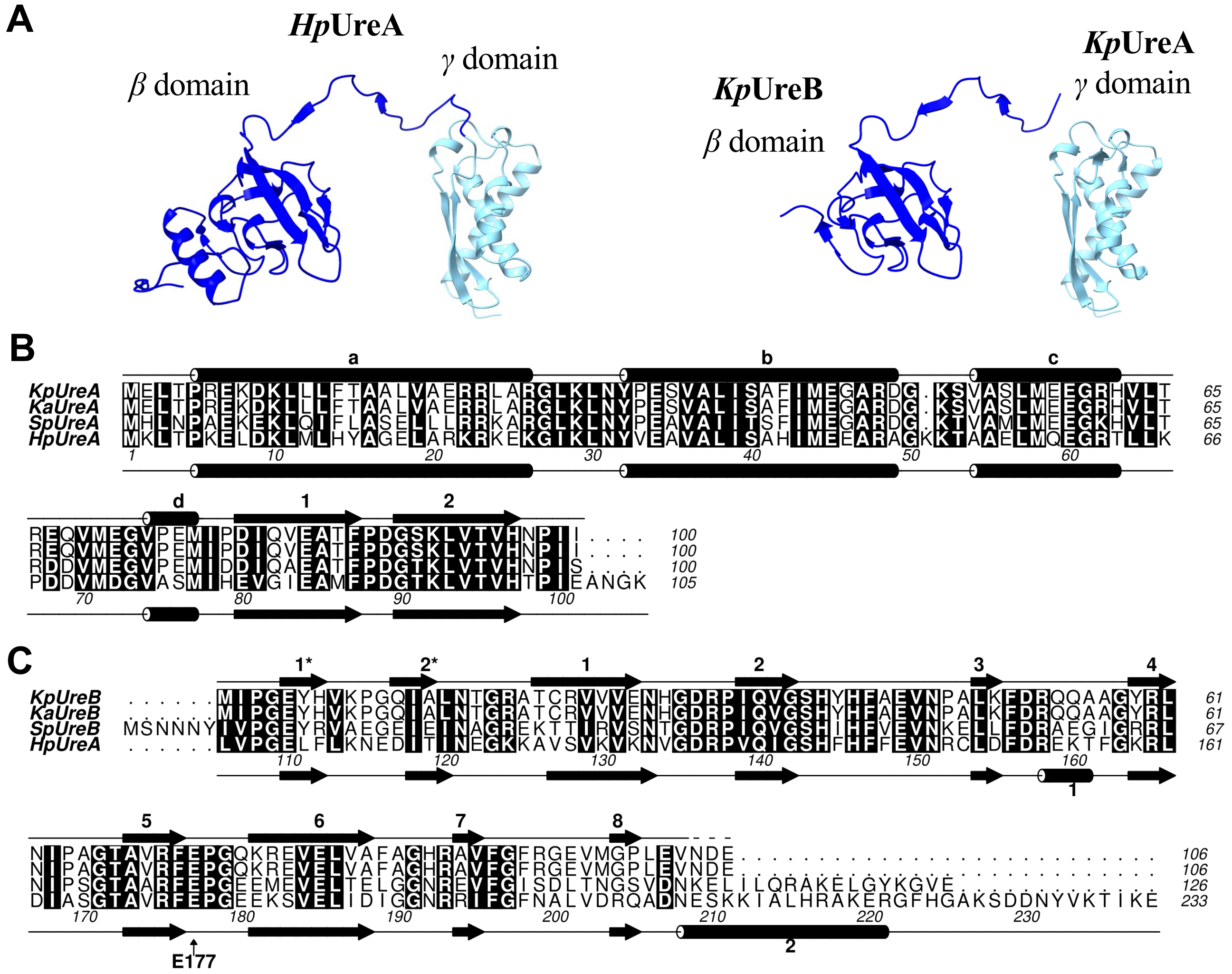

Fig. S4. Structures of the urease β and γ domains in the *Hp*UreFD/urease and *Kp*UreD/urease complexes. (A) Cartoon representation of the structures of the β and γ domains of *H. pylori* and *K. pneumoniae* ureases. In *H. pylori*, the β domain (dark blue) and the γ domain (cyan) are fused together in *Hp*UreA. (B-C) Sequence alignment of γ domain (B) and β domain (C) of *K. pneumoniae (Kp)*, *K. aerogenes (Ka)*, *S. pasteurii (Sp)* and *H. pylori (Hp)* ureases. Secondary structure elements of the *Kp* and *Hp* proteins are indicated above and below the alignment, respectively. Residues that could not be modelled (104-106 of *Kp*UreB) are indicated by dotted lines. The residue numbers of the *H. pylori* sequences are labelled below the alignment. The residues chosen for mutagenesis studies are indicated by arrows.

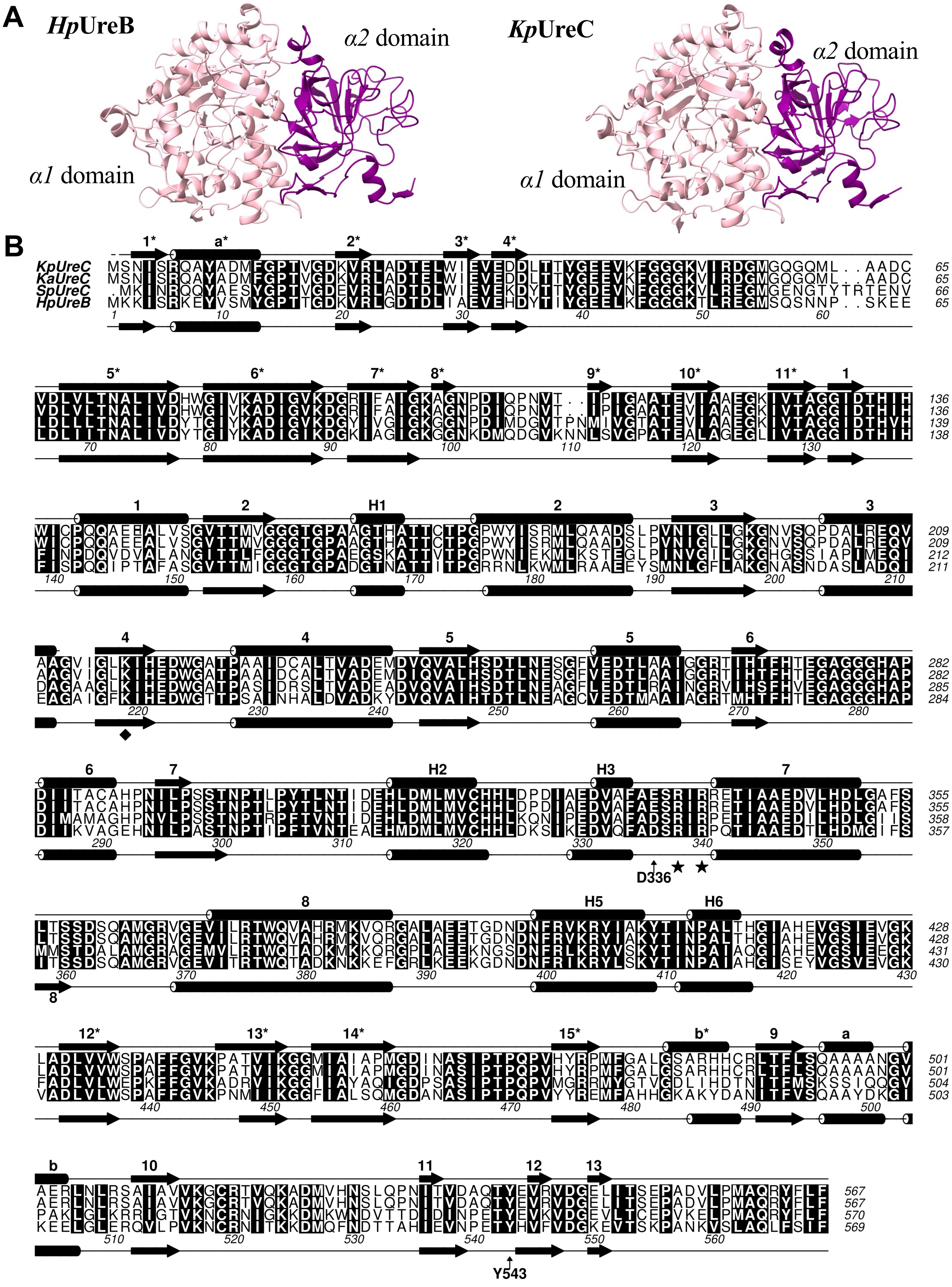

Fig. S5. Structures of the urease α domain in the *Hp*UreFD/urease and *Kp*UreD/urease complexes. (A) Cartoon representation of the structures of the urease α domain (left: *Hp*UreB; right: *Kp*UreC). The α1 domain is coloured in pink, while the α2 domain in purple. (B) Sequence alignment of the α domain of *K. pneumoniae (Kp)*, *K. aerogenes (Ka)*, *S. pasteurii (Sp)* and *H. pylori (Hp)* ureases. Secondary structure elements of the *Kp* and *Hp* proteins are indicated above and below the alignment, respectively. The residue numbers of the *H. pylori* sequences are labelled below the alignment. The two conserved arginine residues (Arg338 and Arg340 of *Hp*UreB) that flip towards UreD upon complex formation are marked by stars. The active site lysine residue (Lys219 of *Hp*UreB) is marked by a diamond. The residues chosen for mutagenesis studies are indicated by arrows.

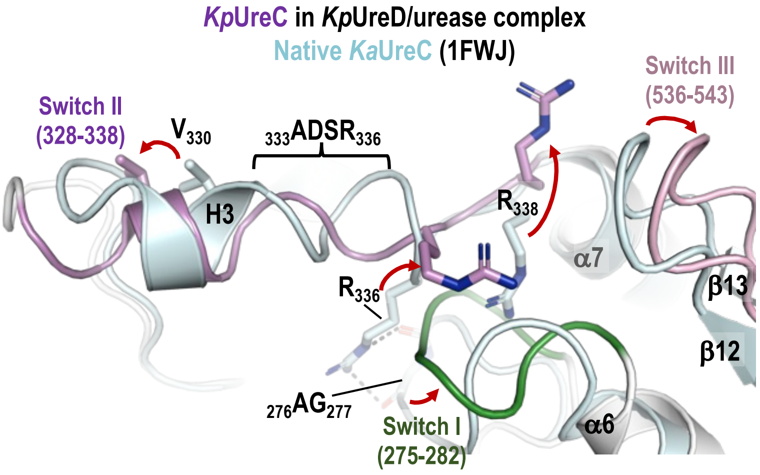

Fig. S6. Structural comparison of *Kp*UreD/urease and native *K. aerogenes* urease. The cryo-EM structure of the *Kp*UreD/urease complex is compared to the crystal structure of native *K. aerogenes* urease (PDB: 1FWJ). Similar conformation changes are observed in the three switch regions of *Kp*UreC upon the formation of *Kp*UreD/urease complex.

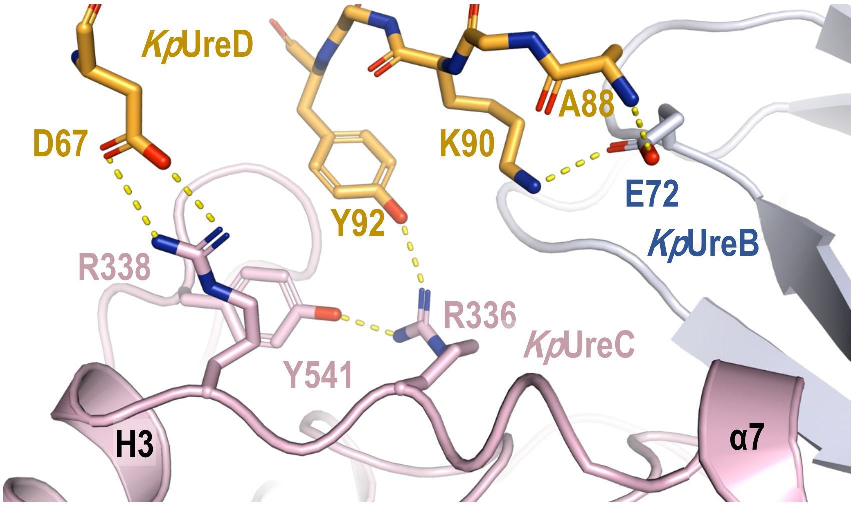

Fig. S7. Interactions between UreD and urease of *Klebsiella pneumoniae* UreD/urease complex. Polar interactions involving conserved residues between *K. pneumoniae* UreD and Urease are indicated as yellow dashes.

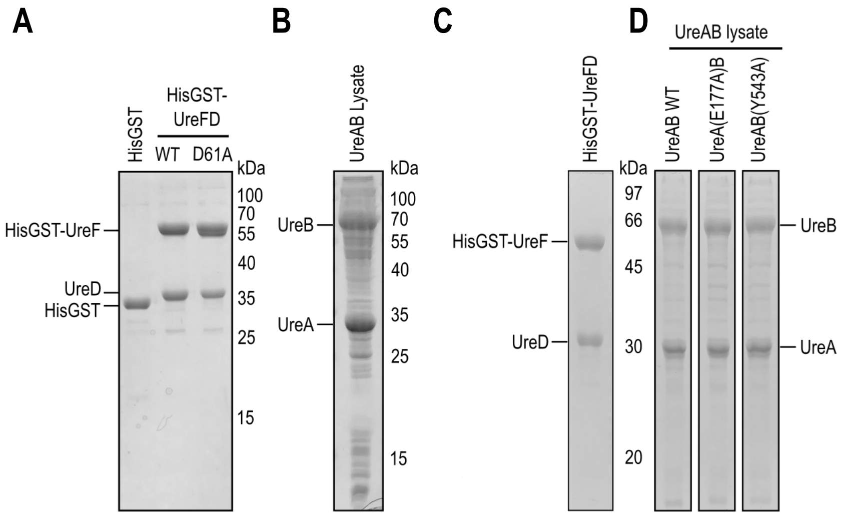

Fig. S8. Protein samples for the pull-down assay testing interactions between *Hp*UreFD and *Hp*UreAB. (A) Wild-type or D61A variant of *Hp*UreD was co-expressed with HisGST-*Hp*UreF and the resulting HisGST-*Hp*UreFD complexes were purified by GST affinity chromatography. HisGST was expressed and purified as a negative control for the pull-down assay. These protein samples were used in the pull-down assay in Fig. 3B. (B) *Hp*UreA and *Hp*UreB were co-expressed in *E. coli*. After sonication, soluble cell lysates of *Hp*UreAB were used in the GST pull-down assay in Fig. 3B. (C) Wild-type *Hp*UreD was co-expressed with HisGST-*Hp*UreF. The resulting HisGST-*Hp*UreFD complex was purified by GST affinity chromatography and used in the pull-down assay in Fig. 3C. (D) E177A and Y543A substitutions were introduced to *Hp*UreA and *Hp*UreB, respectively. Wild-type and variant *Hp*UreAB were expressed in *E. coli*. After sonication, soluble cell lysates of wild-type *Hp*UreAB, *Hp*UreA(E177A)/UreB and *Hp*UreA/UreB(Y543A) were used in the pull-down assay in Fig. 3C.

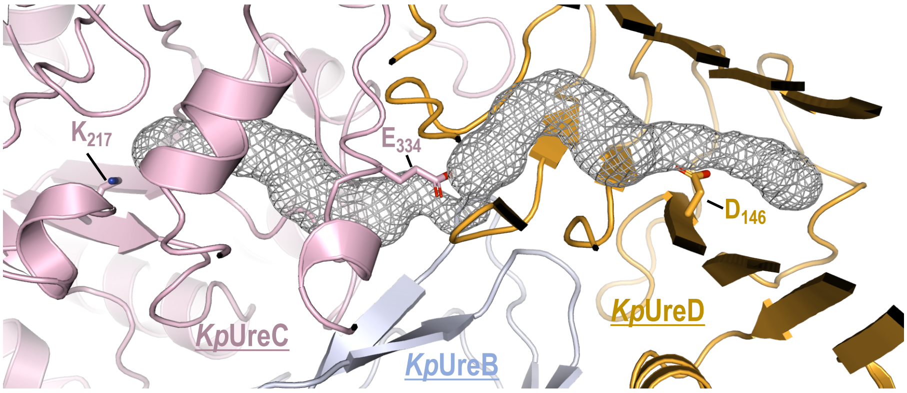

Fig. S9. A similar tunnel exists inside the *Kp*UreD/urease complex. The tunnel reaches the active site residues of Lys217 by passing through conserved regions of *Kp*UreC and *Kp*UreD (Glu334 of *Kp*UreC corresponds to Asp336 of *Hp*UreB; Asp146 of *Kp*UreD corresponds to Glu140 of *Hp*UreD).

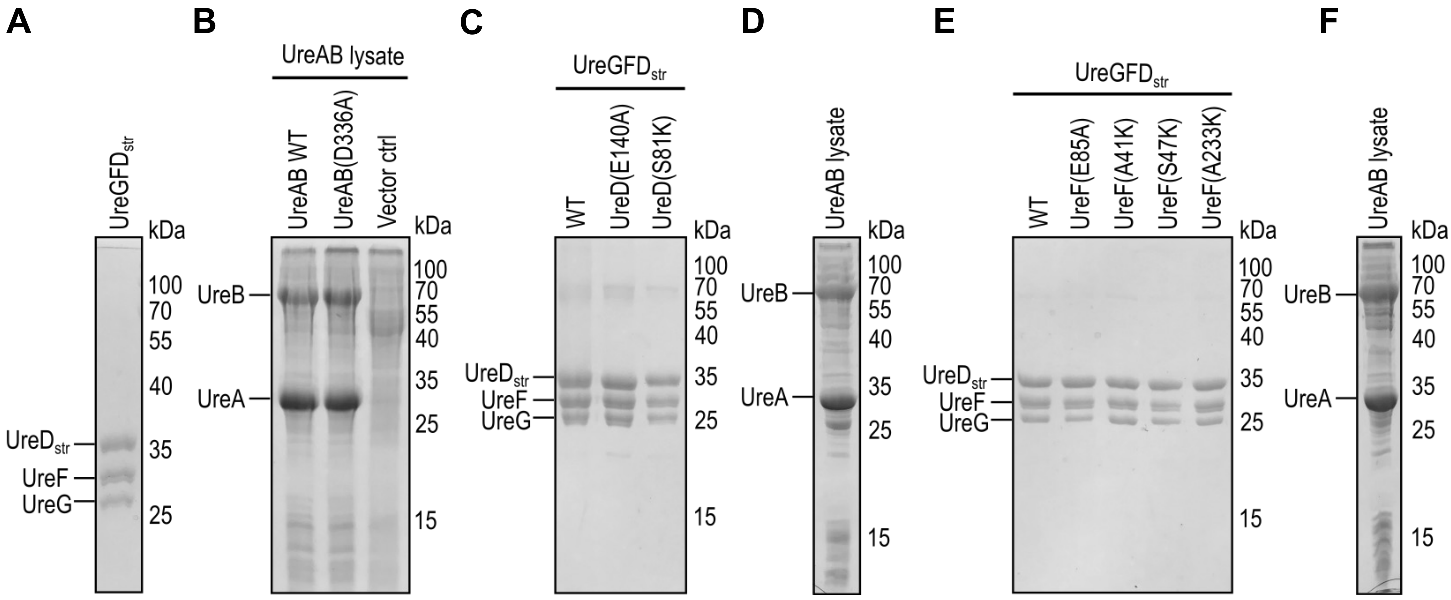

Fig. S10. Protein samples for the pull-down assay testing interactions between *Hp*UreGFD$_{str}$ and *Hp*UreAB. (A, C, E), Strep-tagged *Hp*UreD$_{str}$ (wild-type, or S81K, E140A variant) was co-expressed with (wild-type or A41K, S47K, E85A, A233K variant) *Hp*UreF and *Hp*UreG. The resulting *Hp*UreGFD$_{str}$ complexes were purified by Strep-Tactin affinity chromatography. (B, D, E) *Hp*UreA was co-expressed with (wild-type or D336A variant) *Hp*UreB in *E. coli*. In the vector control, *E. coli* was transformed with the expression plasmid without the coding sequences of *Hp*UreAB. After sonication, soluble cell lysates were used in the pull-down assay. Protein samples in (A)(B), (C)(D), and (E)(F) were used in the pull-down assay presented in Fig. 4D, 4E and 4F, respectively.

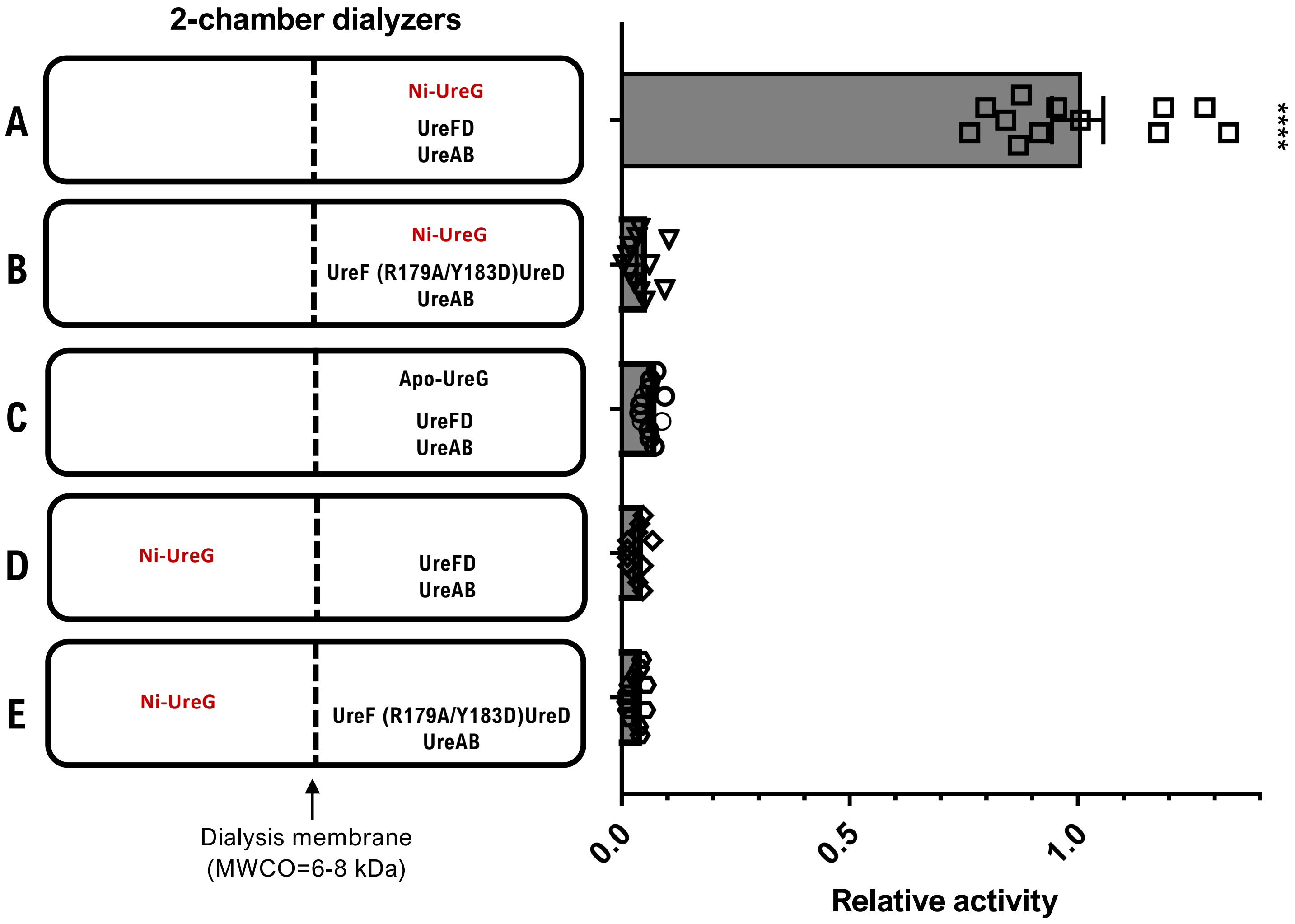

Fig. S11. Urease maturation requires interaction of UreG with the dimeric UreFD. (A-B) To test if the dimerization of UreFD is essential for urease maturation, Ni-bound *Hp*UreG, *Hp*UreFD (WT/variant), *Hp*UreAB were mixed in the same chamber of 2-chamber dialyzers and urease activation was performed as described in the Materials and Methods. The buffer in both chambers contained 20 mM HEPES pH 7.5, 200 mM NaCl, 1 mM TCEP, 2 mM $MgSO_4$ and 1 mM GTP, and the Ni-bound UreG provided the sole source of nickel for urease activation. (C) In the negative control, apo-*Hp*UreG without bound nickel was added instead. Urease activation *in vitro* was abolished in the reaction B using the dimerization-deficient variant (R179A/Y183D). (D-E) To test if direct protein-protein interaction is essential for urease maturation, Ni-bound *Hp*UreG dimer was separated from the rest of the protein components by a dialysis membrane (MW cutoff = 6-8 kDa) in the urease activation assay, which showed that the *in vitro* urease activation was abolished. Error bar represents S.E.M. Activities of reaction B, C, D and E were significantly lower than that of the reaction A (One-way ANOVA, p-value < 0.0001), while there were no significant differences among the activities of reaction B, D, E and the negative control (C).

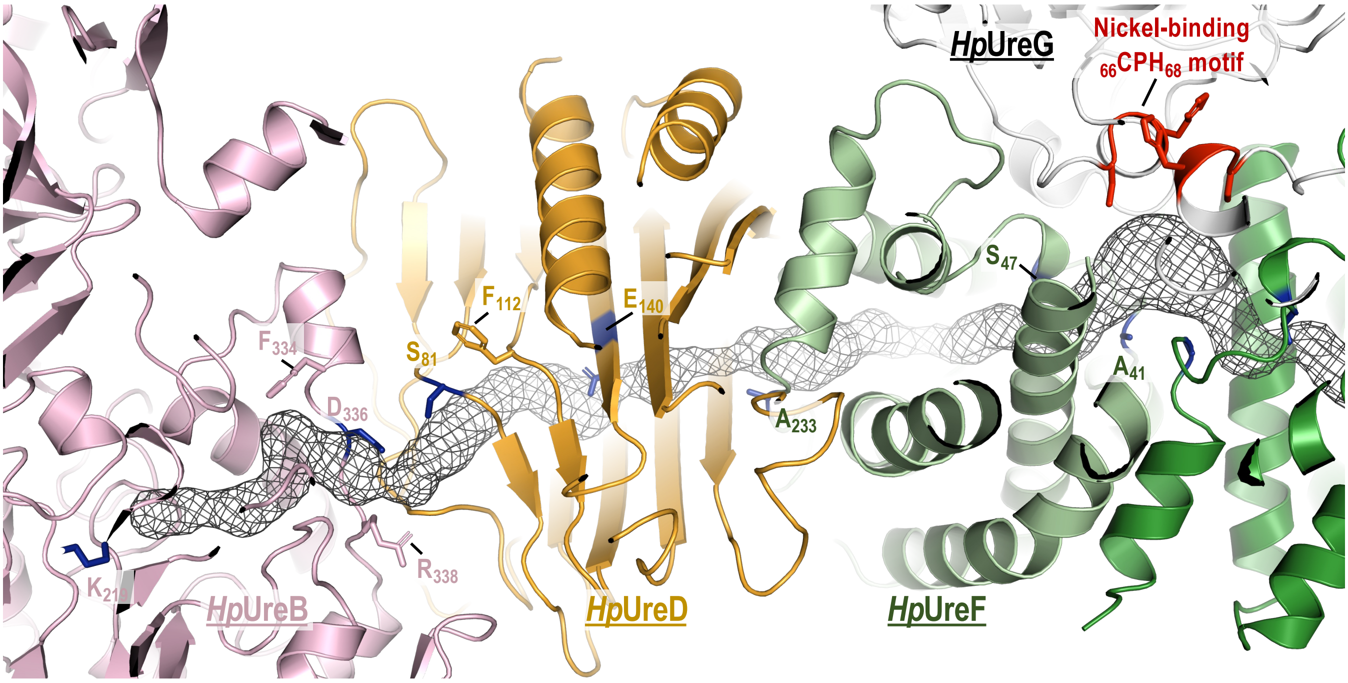

Fig. S12. Modelling of a *Hp*UreGFD/urease complex. The structure of the *Hp*UreGFD complex (PDB: 4HI0) was superimposed onto the *Hp*UreFD/urease complex (this study) using the structure of *Hp*UreF as a reference. Since there is no structural changes in the interface between *Hp*UreD and *Hp*UreF, the final model of the *Hp*UreGFD/urease complex is consisted of the *Hp*UreAB/UreD from the structure of the *Hp*UreFD/urease complex and *Hp*UreF/UreG from the structure of the *Hp*UreGFD complex. It is noted that *Hp*UreG is in the GDP-bound state, where the square-planar coordination for nickel binding of the CPH motif is disrupted so the model is likely representing the structure of the activation complex after GTP hydrolysis. A tunnel (mesh lines) is identified that connects the active site of urease to the nickel-binding site of UreG. The two protomers of the *Hp*UreF dimers are colored in different shades of green.

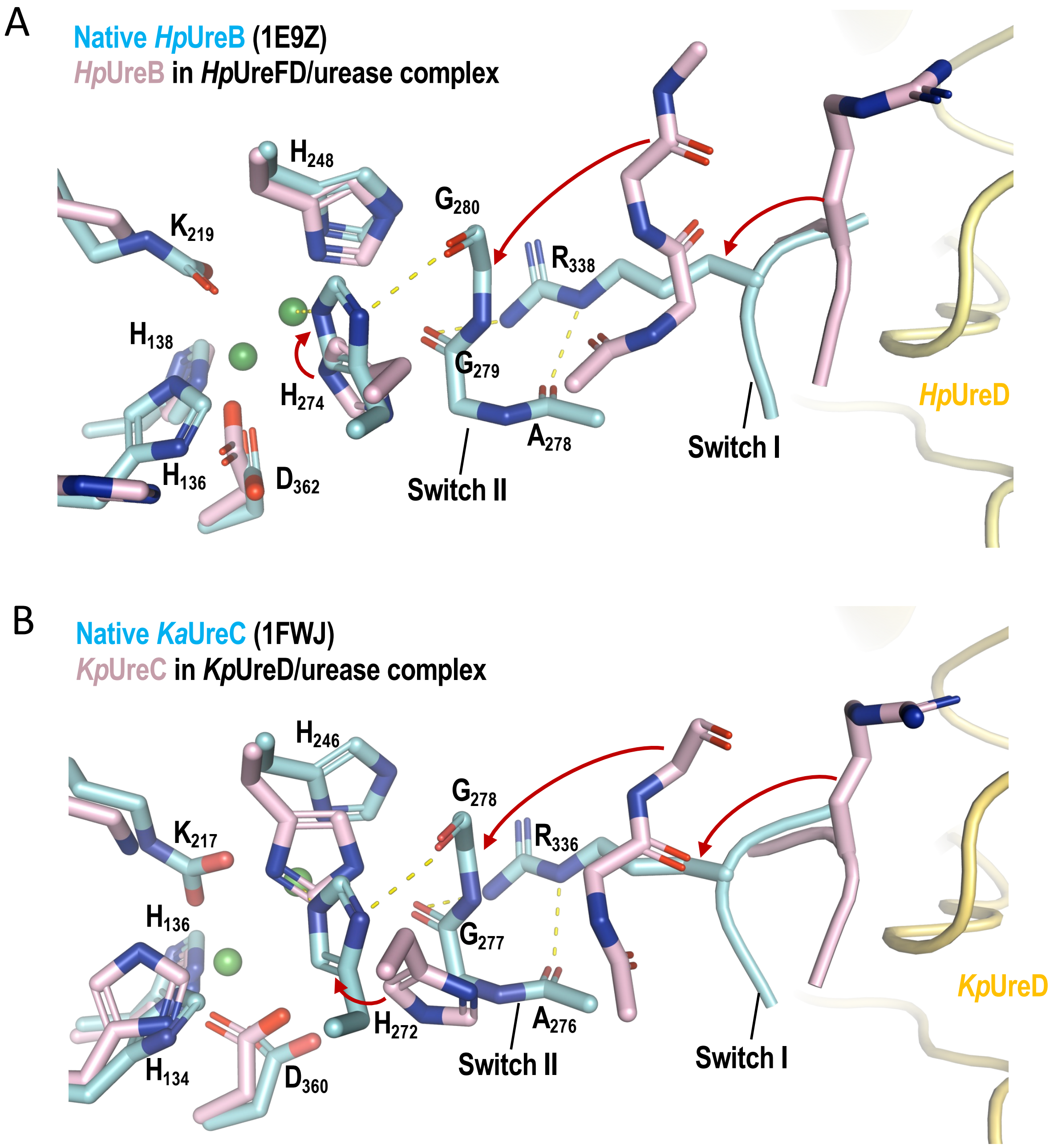

Fig. S13. Nickel binding to the active urease promotes dissociation of UreD. (A) Structure of *Hp*UreB in *Hp*UreFD/urease complex (pink) is compared to that of native *Hp*UreB (cyan). Ni(II) ions are coordinated by carbamylated Lys219 ,His136, His138, His248, His274 and Asp362 in native urease. Nickel binding promotes the formation of a hydrogen bond network (dotted lines) involving His274, Ala278, Gly279, Gly280 and Arg338, inducing conformational changes that promote dissociation of UreD from the activation complex. (B) The hydrogen bond network (dotted lines) that promotes the conformation changes is also conserved in *Klebsiella* urease.

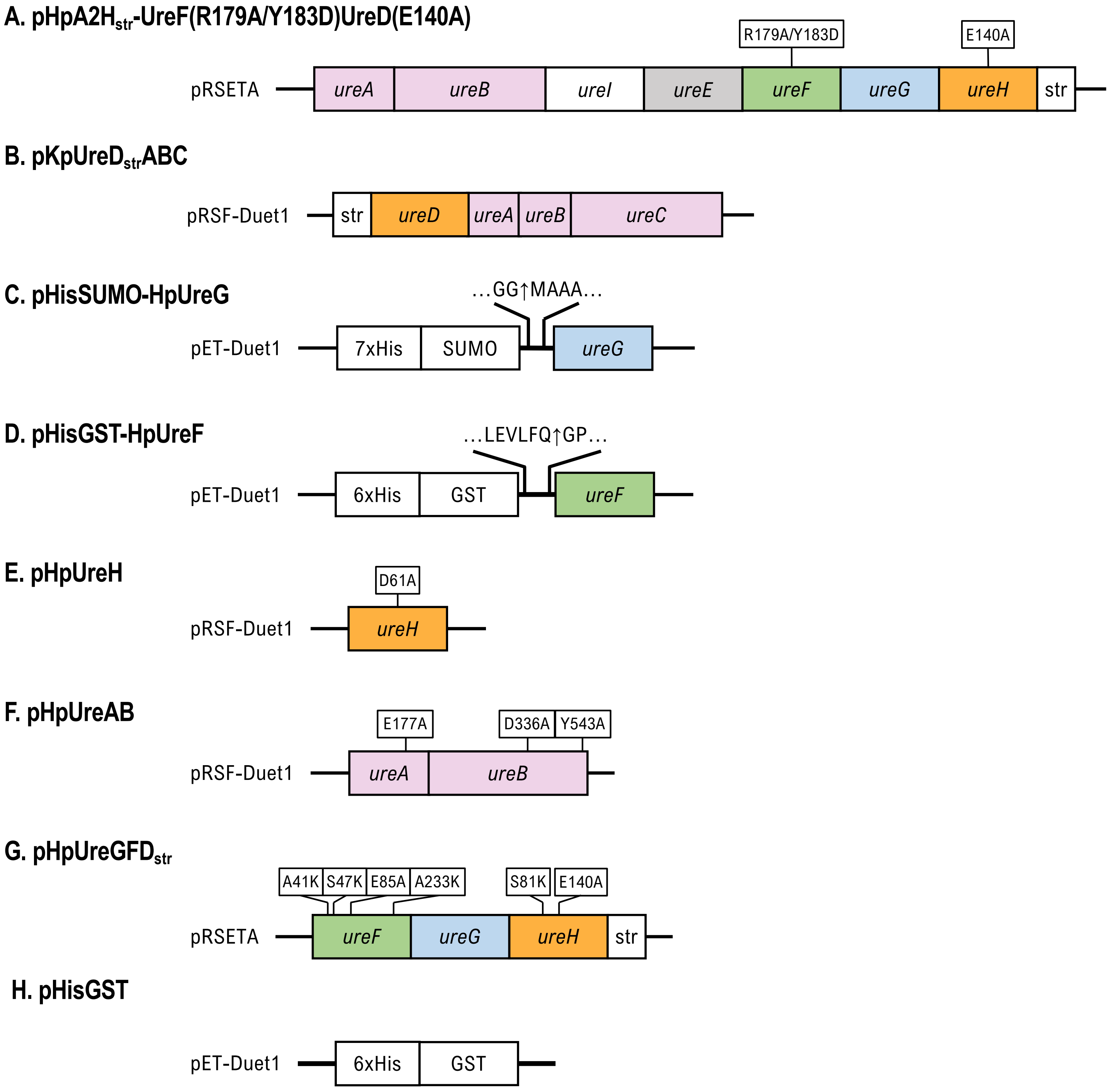

Fig. S14. Summary of expression plasmids used in this study. The names of the parental vector, where the coding sequences are inserted, are indicated on the left. Substitutions to the coding sequences are indicated. It is noted that *H. pylori ureH* gene is an ortholog of *ureD* and encodes the *Hp*UreD protein. str: Strep tag II sequence (WSHPQFEK); SUMO: Small ubiquitin-like modifier protein; GST: Glutathione-S-transferase. The sequences franking the cutting sites (arrows) of SUMO protease (C) and the preScission protease (D) are indicated.

Table S1. Polar contacts between UreD and urease

*Hp*UreD		*Hp*UreA γ domain		Distance (Å)
Residue	Atom	Residue	Atom	
K15	NZ	E101	OE1	2.6
I24	O	R62	NH1	3.0
D26	OD1	Q59	NE2	3.0
N27	ND2	H97	NE2	2.9

*Hp*UreD		*Hp*UreA β domain		Distance (Å)
Residue	Atom	Residue	Atom	
F82	N	E177	OE2	2.9
K84	NZ	E177	OE1	3.2
A148	N	G179	O	3.1
R149	NE	V134	O	3.2
R149	NH2	V134	O	3.2
E244	OE2	S183	N	2.7

*Hp*UreD		*Hp*UreB α domain		Distance (Å)
Residue	Atom	Residue	Atom	
T3	OG1	R340	NH2	3.1
Y4	OH	R340	NH1	2.7
K34	NZ	D316	OD1	3.1
K34	NZ	D316	OD2	3.1
Y40	O	K7	NZ	2.9
E47	OE1	K329	NZ	2.7
R59	NH2	H544	NE2	3.0
D61	OD2	R340	NH1	3.3
D61	OD1	R340	NH2	3.3
Q80	NE2	A335	O	2.4
Q80	NE2	V332	O	2.6
N87	ND2	E541	OE1	3.2
E89	OE2	H544	NE2	2.9

*Kp*UreD		*Kp*UreA γ domain		Distance (Å)
Residue	Atom	Residue	Atom	
L30	O	R61	NH1	3.1
L30	O	R61	NH2	3.2
A31	O	R61	NH1	3.1
R43	NH1	H96	ND1	3.0

K*p*UreD		*Kp*UreB β domain		Distance (Å)
Residue	Atom	Residue	Atom	
A88	N	E72	OE2	3.2
K90	NZ	E72	OE1	3.1
D119	OD2	Q75	NE2	3.0
D242	OD1	K76	NZ	2.7
N243	OD1	K76	N	2.6

*Kp*UreD		*Kp*UreC α domain		Distance (Å)
Residue	Atom	Residue	Atom	
Q42	NE2	D314	OD1	3.2
R43	NH2	D325	OD2	3.4
R43	NE	D325	OD1	2.7
P59	O	R336	N	2.6
D67	OD1	R338	NH2	3.5
D67	OD2	R338	NH1	3.2
K90	NZ	E334	OE2	2.8
Y92	OH	R336	NH1	2.8
R93	NH2	D537	OD1	3.4
R93	NH1	D537	OD2	3.2
R270	NE	E328	OE2	3.3
R270	NH2	E328	OE1	2.4

Table S2. Primers for site-directed mutagenesis

Primer Names	Sequences (5'-3')
HpUreA(E177A)-F	gacagcggtaaggtttgcgcctggcgaagaaaaat
HpUreA(E177A)-R	atttttcttcgccaggcgcaaaccttaccgctgtc
HpUreB(D336A)-F	tcagttcgctgcgtcaaggatccg
HpUreB(D336A)-R	gcggatccttgacgcagcgaactgaa
HpUreB(Y543A)-F	gaaactgcgcatgtgtt
HpUreB(Y543A)-R	aacacatgcgcagtttc
HpUreD(D61A)-F	catgatgaggggcgctgcgcaagatgtgc
HpUreD(D61A)-R	gcacatcttgcgcagcgcccctcatcatg
HpUreD(S81K)-F	cacttcgcaaaaaatttgaaaaaatccataac
HpUreD(S81K)-R	atccttaacttgcaatttg
HpUreD(E140A)-F	gctctatagtgcaatcattgtcg
HpUreD(E140A)-R	aattgagagctagagcgc
HpUreF(A41K)-F	agtcaatgataaagtgttccctattgg
HpUreF(A41K)-R	tgcagaatcagaaattcg
HpUreF(S47K)-F	ccctattggaaaatacacgcattcttttg
HpUreF(S47K)-R	aacaccgcatcattgacttg
HpUreF(E85A)-F	cctttacacggcaatgctgagct
HpUreF(E85A)-R	aactggctagagagattgg
HpUreF(A233K)-F	cctgtgcacgaaaagcgttcaaa
HpUreF(A233K)-R	tggctttcgtctagttctag

Table S3. Urease activity measured in the urease activation assay *

**Urease assay in Fig. 3D**	Specific Activity (μmol NH_3 min^{-1} mg^{-1})	Relative Activity
WT	127.4 ± 3.9	1.00 ± 0.03
Negative Control	12.70 ± 0.12	0.0997 ± 0.0009
UreD(D61A)	17.59 ± 0.15	0.138 ± 0.001

**Urease assay in Fig. 3E**	Specific Activity (μmol NH_3 min^{-1} mg^{-1})	Relative Activity
WT	181.2 ± 2.6	1.00 ± 0.01
Negative Control	14.54 ± 0.89	0.080 ± 0.005
UreA(E117A)	14.07 ± 0.91	0.078 ± 0.005
UreB(Y543A)	9.75 ± 0.51	0.054 ± 0.003

**Urease assay in Fig. 4G**	Specific Activity (μmol NH_3 min^{-1} mg^{-1})	Relative Activity
WT	88.7 ± 2.3	1.00 ± 0.03
Negative Control	6.68 ± 0.22	0.075 ± 0.002
UreB(D336A)	6.83 ± 0.22	0.077 ± 0.002

**Urease assay in Fig. 4H**	Specific Activity (μmol NH_3 min^{-1} mg^{-1})	Relative Activity
WT	41.9 ± 2.5	1.00 ± 0.06
Negative Control	6.45 ± 0.11	0.154 ± 0.003
UreD(S81K)	6.27 ± 0.16	0.150 ± 0.003
UreD(E140A)	6.27 ± 0.09	0.150 ± 0.002

**Urease assay in Fig. 4I**	Specific Activity (μmol NH_3 min^{-1} mg^{-1})	Relative Activity
WT	26.9 ± 1.5	1.00 ± 0.06
Negative Control	5.42 ± 0.27	0.20 ± 0.01
UreF(A41K)	4.80 ± 0.22	0.178 ± 0.008
UreF(S47K)	5.38 ± 0.24	0.200 ± 0.009
UreF(E85A)	6.68 ± 0.21	0.248 ± 0.008
UreF(A233K)	5.85 ± 0.17	0.217 ± 0.006

**Urease assay in Fig. S11**	Specific Activity (μmol NH_3 min^{-1} mg^{-1})	Relative Activity
A. WT UreFD and Ni-UreG in the same chamber	50.84 ± 2.9	1.00 ± 0.06
B. UreF(R179A/Y183D)UreD and Ni-UreG in the same chamber	2.13 ± 0.46	0.042 ± 0.009
C. WT UreFD and apo-UreG in the same chamber	3.15 ± 0.27	0.062 ± 0.005
D. WT UreFD and Ni-UreG in different chambers	1.69 ± 0.26	0.033 ± 0.005
E. UreF(R179A/Y183D)UreD and Ni-UreG in the different chambers	1.55 ± 0.23	0.030 ± 0.005

* Urease activation assays in Fig. 3D, 3E, 4G, 4H, 4I and S11 were performed as described in the Materials and Methods. Specific activities were normalized to those measured for the respective WT controls to obtain the relative activities. Mean and S.E.M. for at least three independent reactions were reported.

Captions for Supplementary Movies

Movie S1. Formation of the *Hp*UreFD/urease induces large conformational changes in *Hp*UreB and *Hp*UreD.

Movie S2. Conformational changes in *Hp*UreB and *Hp*UreD open a tunnel connecting to the urease active site.
